# Protecting the piglet gut microbiota against ETEC-mediated post-weaning diarrhoea using specific binding proteins

**DOI:** 10.1038/s41522-024-00514-8

**Published:** 2024-05-02

**Authors:** Timothy Patrick Jenkins, Norbert Ács, Emma Wenzel Arendrup, Abbie Swift, Ágnes Duzs, Ioanna Chatzigiannidou, Michael Pichler, Tiia Kittilä, Laura Peachey, Lone Gram, Nuria Canibe, Andreas Hougaard Laustsen, Susanne Brix, Sandra Wingaard Thrane

**Affiliations:** 1 Department of Biotechnology and Biomedicine, Technical University of Denmark, Kongens Lyngby, Denmark; 2 Bactolife A/S, Rønnegade 8, Copenhagen, Denmark; 3 Veterinary Sciences, University of Bristol, Bristol, UK; 4 Department of Animal Science, Aarhus University, Tjele, Denmark

**Keywords:** Health care, Pathogens, Applied microbiology

## Abstract

Post-weaning diarrhoea (PWD) in piglets presents a widespread problem in industrial pig production and is often caused by enterotoxigenic *E. coli* (ETEC) strains. Current solutions, such as antibiotics and medicinal zinc oxide, are unsustainable and are increasingly being prohibited, resulting in a dire need for novel solutions. Thus, in this study, we propose and evaluate a protein-based feed additive, comprising two bivalent heavy chain variable domain (V_H_H) constructs (V_H_H-(GGGGS)_3_-V_H_H, BL1.2 and BL2.2) as an alternative solution to manage PWD. We demonstrate in vitro that these constructs bind to ETEC toxins and fimbriae, whilst they do no affect bacterial growth rate. Furthermore, in a pig study, we show that oral administration of these constructs after ETEC challenge reduced ETEC proliferation when compared to challenged control piglets (1-2 log_10_ units difference in gene copies and bacterial count/g faeces across day 2–7) and resulted in week 1 enrichment of three bacterial families (*Prevotellaceae* (estimate: 1.12 ± 0.25, *q* = 0.0054)*, Lactobacillaceae* (estimate: 2.86 ± 0.52, *q* = 0.0012), and *Ruminococcaceae* (estimate: 0.66 ± 0.18, *q* = 0.049)) within the gut microbiota that appeared later in challenged control piglets, thus pointing to an earlier transition towards a more mature gut microbiota. These data suggest that such V_H_H constructs may find utility in industrial pig production as a feed additive for tackling ETEC and reducing the risk of PWD in piglet populations.

## Introduction

An ever-increasing amount of meat is being consumed globally, largely due to massive population growth, with the world population expected to rise to 9.7 billion by 2050^1^. Pigmeat has become the most consumed meat, closely followed by poultry. Indeed, the global per capita consumption of pigmeat increased from 8.0 kg in 1961 to 15.6 kg in 2019^2^. Current industrial practices require suckling piglets to be separated from the sow at an early age, i.e., during their third or fourth week of life. However, the subsequent stress of the changed environment, the loss of maternal antibodies present in the sow’s milk, and the underdeveloped immune system of the piglet, alongside an immature gut microbiota, leads to post-weaning diarrhoea (PWD) and other health issues. PWD prevalence in farms vary (20–50%)^3,4^ depending on the country and the disease history of the herd.

PWD is a multifactorial disease, but it is commonly associated with enterotoxigenic *E. coli* (ETEC) strains, which exploit the compromised immune system of the piglets to establish themselves in the gut by adhering to intestinal enterocytes via their fimbriae^5,6^. These pathogens subsequently produce a range of enterotoxins, including heat-labile (LT) and heat-stable (ST) enterotoxins. This results in diarrhoea, causing the piglets to suffer from severe dehydration, weight loss, and, in severe cases, death^5^, impacting both animal productivity and welfare.

ETEC pathotypes are distinguished based on their fimbriae and toxin gene repertoire, and with a high frequency of horizontal gene transfer due to selective pressure imposed by antimicrobials, pathotypes are ever-mutating. The most abundant virulence factors found in porcine ETEC pathotypes are F4 and F18 (fimbriae), and LT and STa/STb (toxins)^3,7^. To date, two efficacious veterinary drugs have been applied for the management of ETEC-related PWD, i.e., antibiotics and medicinal zinc oxide. However, the use of antibiotics is unsustainable due to antimicrobial resistance development with subsequent animal and human health impacts. Additionally, some resistance genes do not govern resistance to just one class of antibiotics, and acquisition and spread of of those genes in the population may therefore lead to multidrug resistance, which is a huge burden on veterinary care and healthcare from a One Health perspective^8–10^. Medicinal zinc oxide has beneficial effects on reducing PWD, while increasing feed intake and weight gain^11,12^. However, most of the zinc oxide included in the feed is excreted and not metabolised, leaving high concentrations in manure causing heavy metal pollution of the environment^13^.

Notably, antibiotic usage in animal production is increasingly restricted, with growth promoters being banned in the EU since 2006^14^. Similarly, medicinal zinc has been banned from use in the EU as of 2022^14,15^. To manage PWD, farmers employ several other products and practices, such as optimising the feed composition via the introduction of feed additives claimed to support piglet gut health and nutrition status, *e.g*., organic acids, prebiotics, probiotics, and essential oils^16–18^. Despite the wide range of available feed additives, PWD remains a significant problem in industrial pig production.

This study outlines the further development and testing of a protein-based feed additive, comprising bivalent V_H_H constructs, which are dimeric protein constructs comprising two V_H_Hs joined by a protein linker, for precision management of ETEC-mediated PWD. The V_H_H constructs emulate the natural lactogenic immunity of the piglets by binding to, and blocking of, ETEC virulence factors in the absence of maternal antibodies. The aim of this study was to validate the application of the proteins in vivo, at industrially relevant inclusion rates, and to examine the synergistic effect of combining protein constructs blocking both toxins and adhesins simultaneously on gut microbiota stability and composition. Previously, we demonstrated in a proof-of-concept study that a bivalent V_H_H construct (BL1.2) derived from camelid antibodies is stable under industrially relevant conditions and can prevent the proliferation of ETEC in weaned piglets^19^. We identified high-affinity binding between the product and the target virulence factor, the porcine ETEC F4 type fimbriae, for one bivalent V_H_H construct. Taking into consideration the diversity of virulence factors expressed by porcine ETEC strains, we hypothesised that specific blocking of both ETEC fimbriae and toxins, using a combination of bivalent V_H_H constructs, could provide synergy for better management of PWD caused by diverse ETEC strains.

**Fig. 1:**
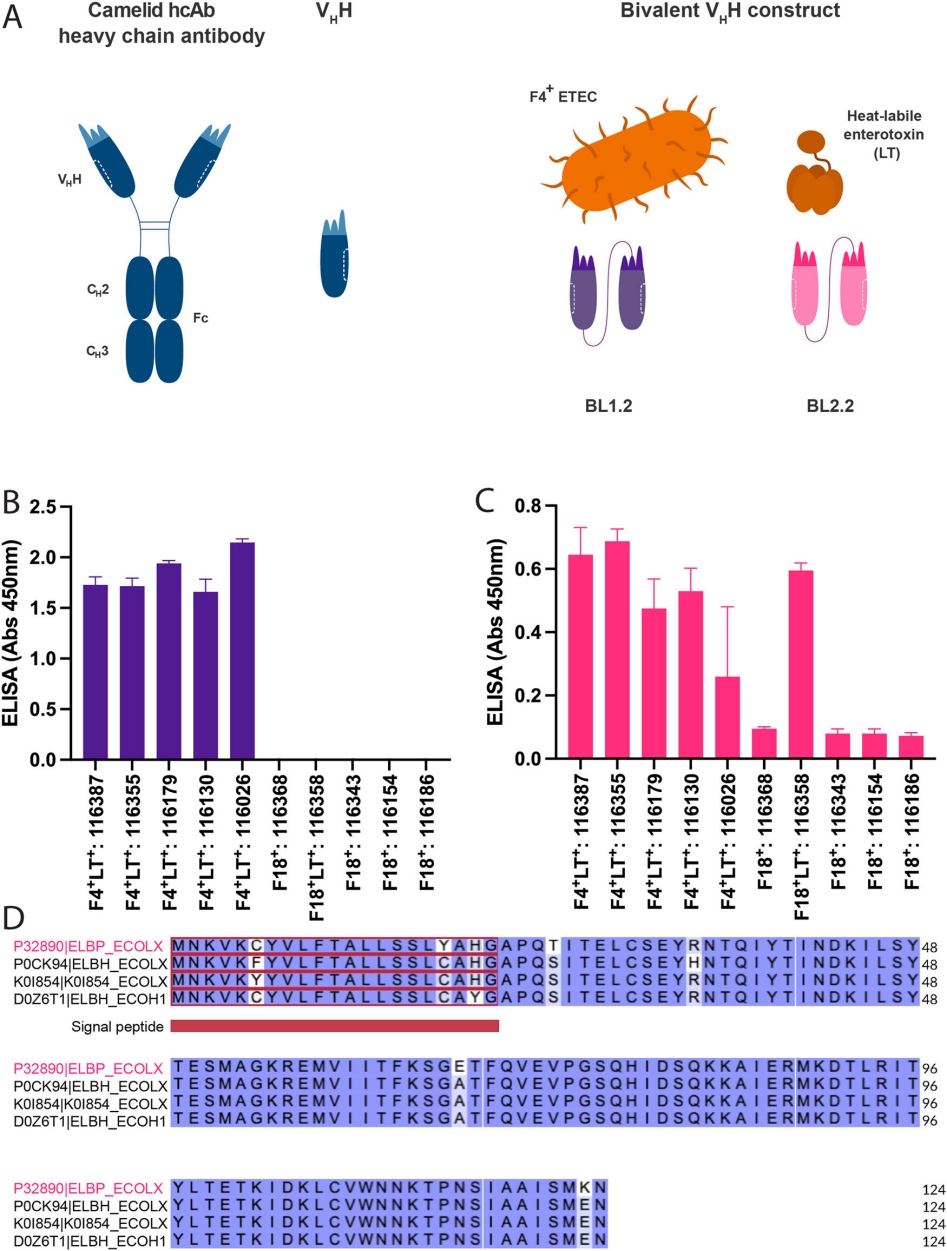
Design overview of V_H_H constructs and robust binding of BL1.2 and BL2.2 to a range of native F4 fimbriae and LT expressed by wild-type porcine ETEC strains. **A** The VHH constructs used in this study are based on single-domain antibodies derived from camelid heavy chain antibodies, with BL1.2 designed to inhibit F4^+^ ETEC adhesion by blocking its fimbriae, and BL2.2 designed to inhibit excreted LT^+^ toxins. **B** ELISA showing binding of BL1.2 to F4 fimbriae from different F4^+^ ETEC strains from Danish farms. **C** ELISA showing binding of BL2.2 to LT toxins in the supernatant of F4^+^LT^+^ ETEC and F18^+^LT^+^ ETEC strains from Danish farms. **D** Amino acid sequence alignment of LT toxins from porcine (magenta) and human (black) pathogenic ETEC strains.

Here, we report the development of a new specific bivalent V_H_H construct (BL2.2) that neutralises *E. coli* LT by inhibiting the toxin’s ability to bind the monosialotetrahexosylganglioside (GM1) gut receptor. We further demonstrate, for the first time, how the oral administration of a combination of bivalent V_H_H constructs (BL1.2 and BL2.2, Fig. 1A), binding F4 fimbriae and LT from porcine ETEC strains, respectively, reduces the proliferation of F4^+^ ETEC in the gastrointestinal (GI) tract of piglets. Finally, our results indicate that feeding of V_H_H constructs may improve piglet health in a synergistic manner, consequently strengthening the piglet microbiota against fluctuations and ensuring quicker maturation.

## Results

### Bivalent V_H_H construct binds ETEC-derived LT

In a previous study, it was shown that BL1.2 binds F4 fimbriae with high specificity and affinity^19^. BL2.2 was constructed like BL1.2, as a bivalent V_H_H construct composed of two identical V_H_Hs, connected via a (GGGGS)_3_ linker, but with specificity towards the B-subunit of LT (Fig. 1A). The BL.2.2 V_H_H was previously discovered using commercially acquired LT, originating from a human pathogenic ETEC strain^20^. We tested the in vitro ability of BL1.2 and BL2.2 to bind to various pathogenic porcine F4^+^ and F18^+^ ETEC strains from Danish farms of which some ETEC strains can produce LT (LT^+^). BL1.2 showed robust binding across the F4^+^ ETEC strains, while no binding was detected towards ETEC F18^+^ fimbriae strains (Fig. 1B). BL2.2 was demonstrated to bind to LT expressing F4^+^ and F18^+^ ETEC strains (Fig. 1C). As BL2.2 was generated against a human pathogenic LT-producing ETEC strain, this latter finding might be due to the high sequence conservation between LT toxins from porcine and human pathogenic ETEC strains (Fig. 1D). BL1.2 and BL2.2 were tested for thermal stability in PBS (pH 7.2) to verify their stability and applicability. Based on the Protein Thermal Shift™ assay (Thermo Scientific), the unfolding temperature (Boltzmann T_m_) of BL1.2 and BL2.2 is 74 and 54 °C respectively (Supplementary Figure 1).

### Bivalent V_H_H constructs do not affect the growth rate of porcine F4^+^LT^+^ ETEC strains

**Fig. 2:**
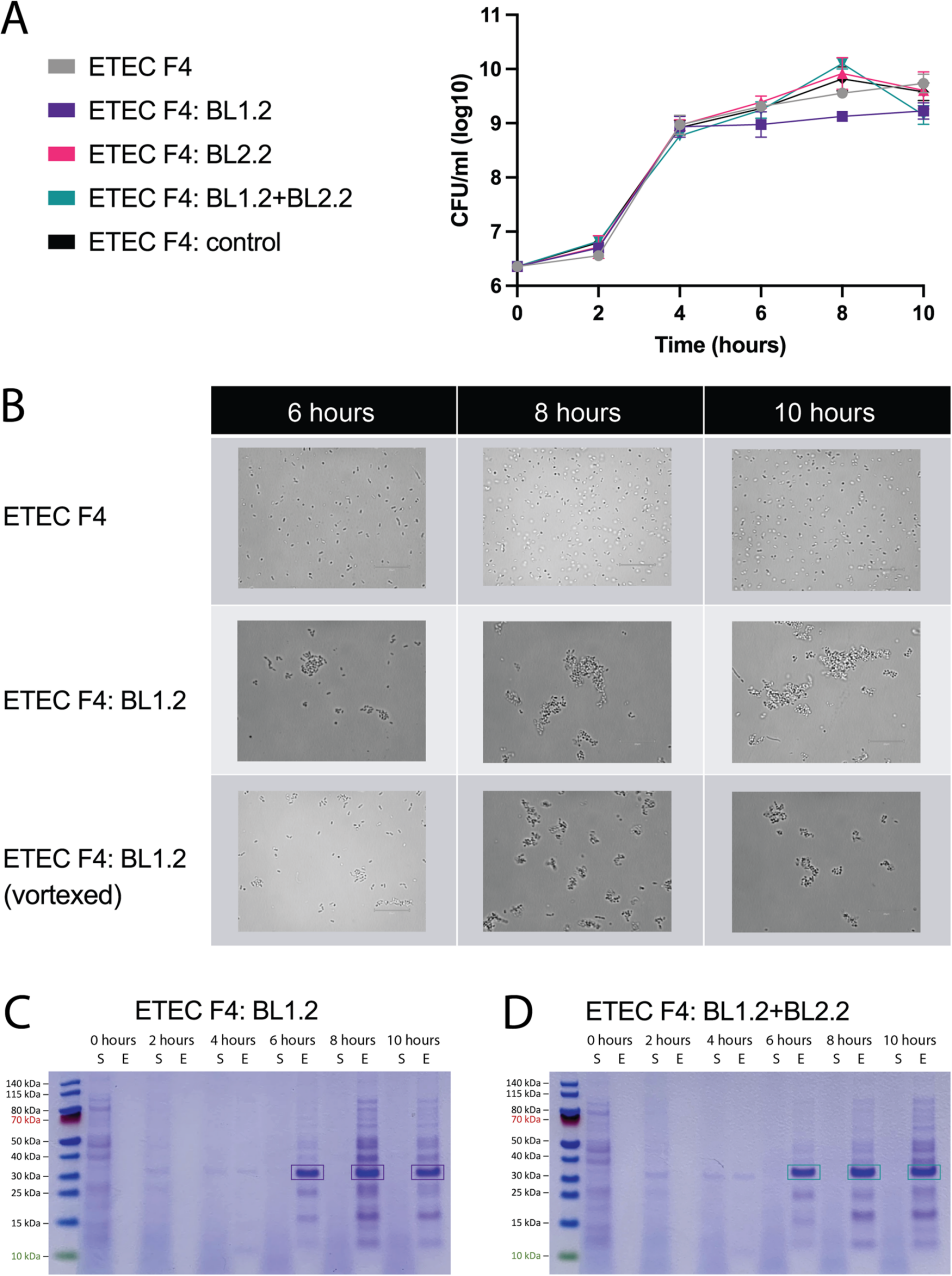
F4^+^LT^+^ ETEC growth in the presence of bivalent V_H_H constructs. **A** Growth of F4^+^LT^+^ ETEC in the presence of bivalent V_H_H constructs targeting F4 fimbriae (BL1.2), LT (BL2.2), an unspecific control construct or no construct visualized as log transformed CFU/mL. Data are based on technical replicates from one biological replicate of each treatment and represented as mean (of log transformed values) with standard error of the mean (SEM). No statistically significant differences were found using two-factor ANOVA. **B** Microscopy pictures of the ETEC cells grown with and without BL1.2 showed increasing cell aggregates in presence of BL1.2 in accordance with incubation time. Samples with BL1.2 were also vortexed and observed. Scale bar in lower left corner respresents 20 μm. **C**, **D** SDS-PAGE visualisation of recovered BL1.2 from the supernatant (S) and eluate (E) recovered upon weak acid treatment of samples (BL1.2 and BL2.2 will migrate at a size of 28 kDa). V_H_H constructs bound to F4 fimbriae during F4^+^LT^+^ ETEC growth experiment will be present in the eluate (E). An increase in bound BL1.2 with increasing bacterial growth was observed. See also Supplementary Fig. 2.

BL1.2 was designed to block the adhesion of F4^+^ ETEC strains to the intestinal wall, while BL2.2 was designed to neutralise LT toxins secreted from LT^+^ ETEC strains. Neither bivalent V_H_H constructs were, thus, designed to affect the growth of the bacteria. To confirm the absence of such effects, an F4^+^LT^+^ ETEC strain was grown in the absence or presence of either BL1.2, BL2.2, both BL1.2 and BL2.2, or a control bivalent V_H_H construct. Growth was followed by quantification of colony-forming units (CFU) and *E. coli* grew with similar growth patterns across study groups (Fig. 2A). Despite the similar growth pattern, it was demonstrated using microscopy that BL1.2 addition resulted in the formation of bacterial aggregates (Fig. 2B), probably facilitated by the ability of BL1.2 to cross-link bacteria as previously reported^19^. The aggregates were stable and did not disintegrate by mechanical force (Fig. 2B). To ensure that BL1.2 remained bound to the F4 fimbriae of F4^+^LT^+^ ETEC bacteria throughout the experiments, it was investigated whether bound bivalent V_H_H construct could be recovered from the cells at different time points, following weak acid treatment. Using SDS-PAGE, the recovered BL1.2 was detected (Fig. 2C and Fig. 2D), and band intensity appeared to increase as CFU increased, indicating that the amount of bound BL1.2 to the F4 fimbriae increased with bacterial growth. The band at ~28 kDa was not detected for any of the other experimental groups (Supplementary Fig. 2).

### Oral application of bivalent V_H_H constructs reduces ETEC proliferation

**Fig. 3:**
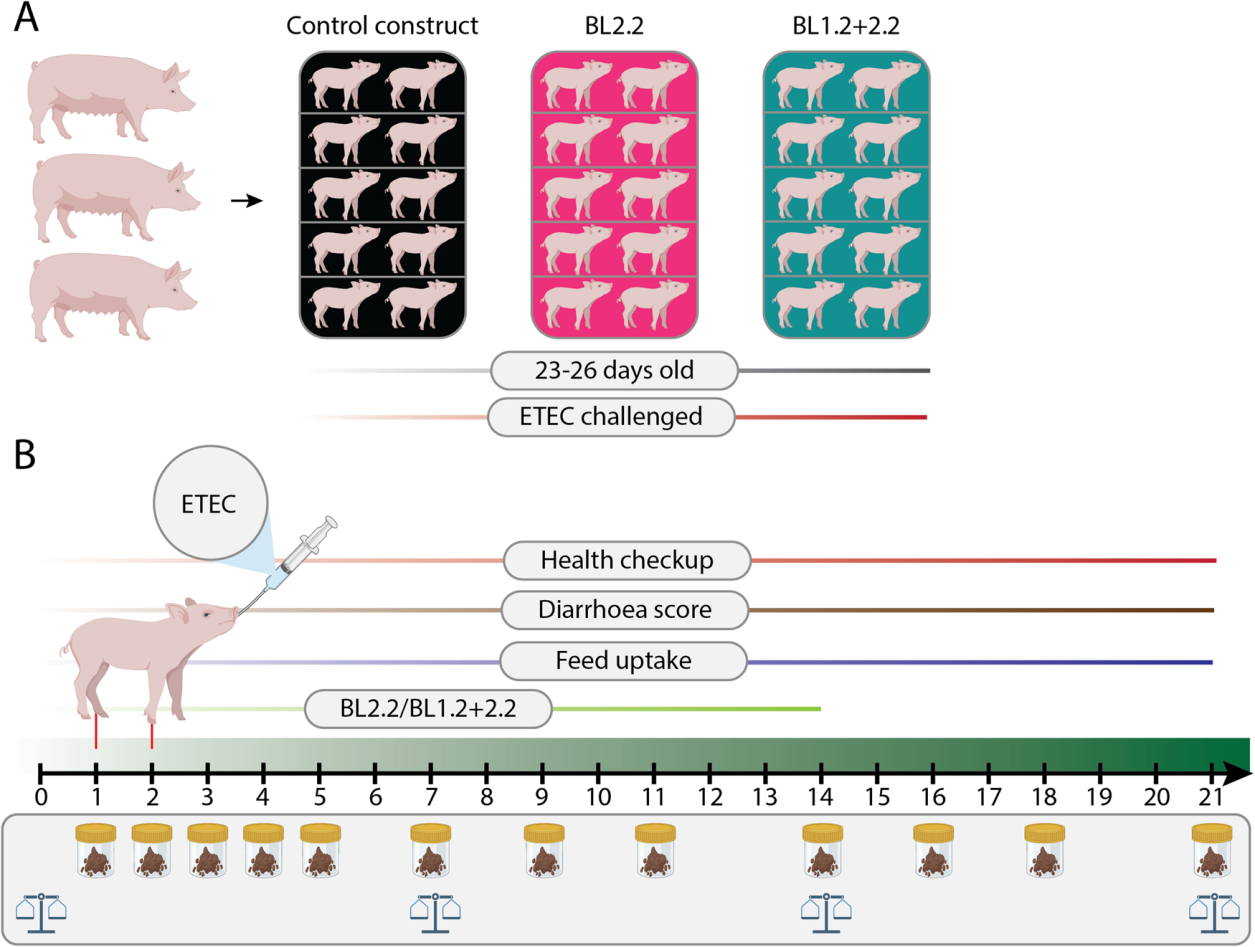
Schematic illustration of experimental outline, timeline, and sampling. **A** The study included 30, 23–26-day-old piglets from three sows divided into three groups (*n* = 10): piglets receiving either nonspecific V_H_H Control; an ETEC LT^+^-targeting bivalent V_H_H construct (BL2.2); or an ETEC F4^+^ fimbriae and LT^+^ targeting bivalent V_H_H construct (BL1.2 + BL2.2) for two weeks. On days 1 and 2 after weaning, all pigs were orally challenged with an ETEC F4^+^LT^+^ strain. **B** Throughout the study, the piglets’ health condition, body weight, and feed intake were recorded; faecal samples were collected at specific timepoints.

To test the efficacy of the constructs in vivo, a study with newly weaned (23–26 days of age) piglets (*n* = 30) of both sexes from 3 sows (tested homozygous carriers of the dominant gene encoding intestinal ETEC F4 fimbriae receptors) was conducted (Fig. 3A). Three experimental groups were examined, i.e., Control (non-specific construct; *n* = 10), BL2.2 (*n* = 10), and BL1.2 + BL2.2 (*n* = 10). Groups BL2.2 and BL1.2 + BL2.2 received 4.8 mg of the respective bivalent V_H_H construct solution orally, suspended in PBS to a total volume of 5 mL, with a few drops of added apple juice twice a day, starting on the weaning day (day 0) and continued until day 14. Further, an Advance Milky Flavor (Luctarom Advance) was included in the feed, to encourage piglets to start eating. On days 1 and 2 of the experiment, all piglets received 5 mL of the AUF4 F4^+^LT^+^ ETEC strain (10^9^ CFU/mL)^19^. The piglets had access to creep feed from day 14 after birth and were fed *ad libitum* with the same mixture throughout the study period. The study lasted 21 days (Fig. 3B).

**Fig. 4:**
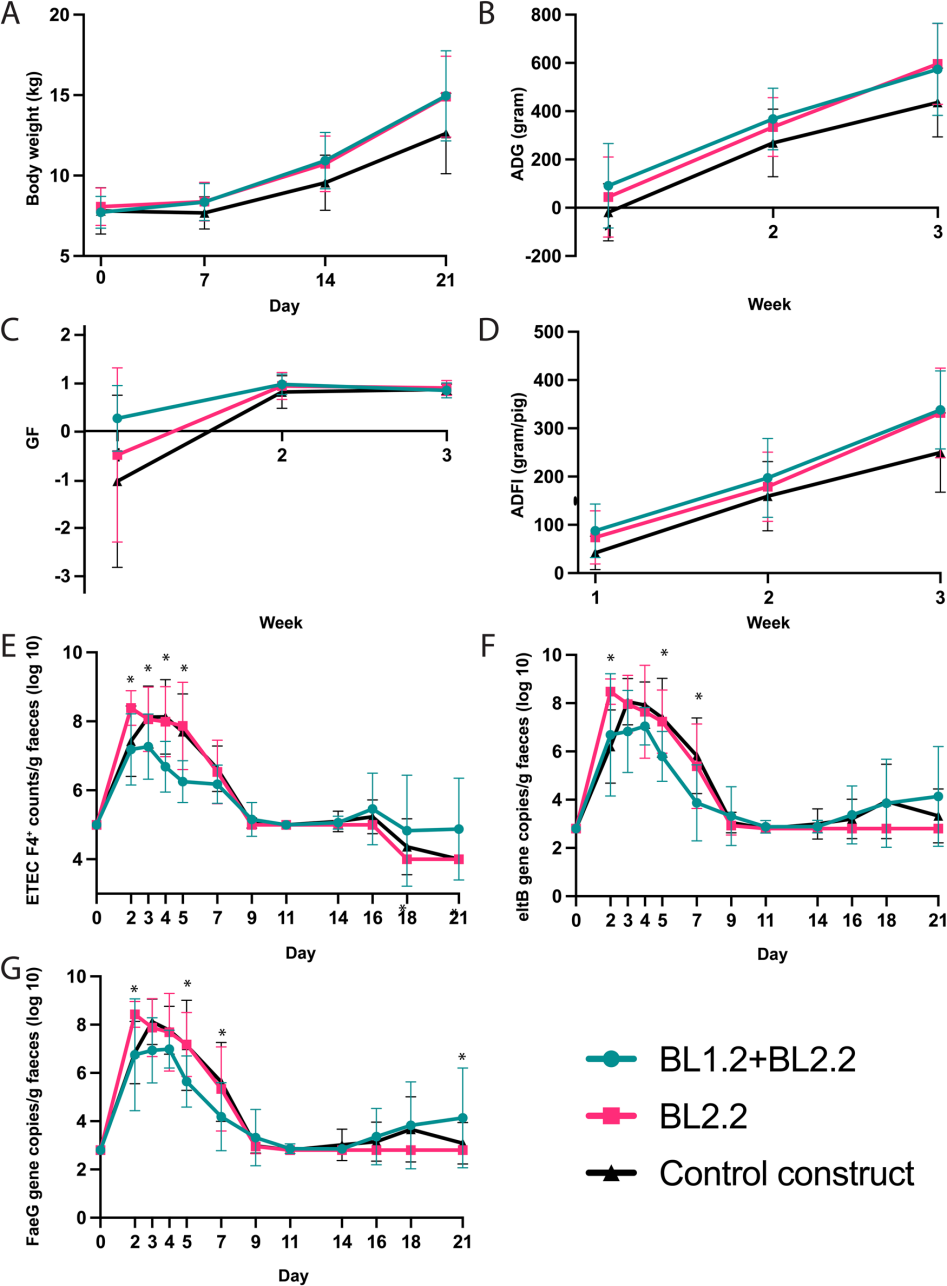
Piglet performance throughout the 21-day study. **A** Their body weight, (**B**) average daily gain (ADG), (**C**) growth-to-feed ratio (GF), (**D**) average daily feed intake (ADFI), (**E**) number of ETEC F4^+^ log CFU/g faeces, (**F**) *eltB* (LT toxin) gene copy number/g faeces in the experimental groups, and (**G**) *faeG* (F4ac fimbriae) gene copy numbers/g faeces were monitored throughout the study. Centre line corresponds to mean, bounds of box to lower and upper quartile and whiskers correspond to min and max. Error bars represent mean ± SD. Asterisks indicate a significant difference. Differences were assessed using a linear mixed effects model. Treatment was a fixed effect, while initial body weight was included as a covariate for ADG and ADFI. Pen and sow effects were considered as random effects. Assumptions of normality and homogeneity of variance were confirmed. Satterthwaite approximation was used for denominator degrees of freedom. Normal or lognormal linear mixed effects model was used to test results from faecal samples with treatment, day (as categorical variable) and their interaction as fixed effects. Pen and sow were considered as random effects. A continuous-time autoregressive covariance structure of order 1 was used to account for correlation among measures from the same pig. Differences between means were compared pairwise using *P*-values adjusted for multiple comparisons using the Holm–Bonferroni adjustment. *P < 0.05. See also Supplementary Fig. 3.

The impact of the V_H_H constructs on the piglets’ growth performance was not statistically significant. Numerically improved values were observed, though. As the study progressed, there were increasing numerical (*P* = 0.17), differences in piglet weight between the two test groups (BL2.2 and BL1.2 + BL2.2) and the Control (Fig. 4A). After the first week, piglets in the BL2.2 and BL1.2 + BL2.2 groups weighed an average of 700/680 g more than the Control group; after two and three weeks they weighed 1.17/1.38 kg and 2.29/2.24 kg more than the Control group. These numerical improvements were also reflected in their average daily gain (ADG; Fig. 4B), growth:feed (GF; Fig. 4C) ratio, and average daily feed intake (ADFI; Fig. 4D) where BL1.2 + BL2.2 piglets ate 2.4 kg more throughout the study than the Controls. No impact could be observed in the piglets’ diarrhoea prevalence based on the faecal scores or faecal dry matter percentage (Supplementary Fig, 3A, B).

Faecal counts of ETEC F4^+^ across the study groups varied significantly (Fig. 4E). While faecal shedding was similar across BL2.2 and Control piglets, it was significantly lower in BL1.2 + BL2.2 piglets on days 3, 4, and 5 compared to the Control group (*P* = 0.041, 0.0002, and <0.0001) and days 2, 3, 4, and 5 compared to the BL2.2 group (*P* = 0.002, 0.04, 0.0005, and <0.0001; Fig. 4E). Furthermore, shedding was also higher (*P* = 0.03) in the BL2.2 group compared to Control piglets on day 2.

Quantitative (q)PCR analyses further revealed similar patterns for the level of *eltB* gene (LT2 toxin; Fig. 4F) and *faeG* (F4; Fig. 4G) genes in BL2.2 and Control piglets, while significantly fewer were detected in BL1.2 + BL2.2 piglets. For the *faeG* gene, these differences were significant on days 5 and 7 compared to Control piglets (*P* = 0.02 and 0.007) and on days 2, 5, and 7 compared to BL2.2 piglets (*P* = 0.01, 0.03, and 0.04). For *eltB*, significantly lower levels were recorded in BL1.2 + BL2.2 piglets when compared to the Control group on days 5 and 7 (*P* = 0.03 and 0.001). On days 2, 5, and 7, *eltB* gene levels were also significantly lower compared to BL2.2 piglets (*P* = 0.01, 0.05, and 0.007).

At the conclusion of the study, the stability of binding protein BL1.2 when subjected to prolonged exposure to the stomach content of live piglets was tested. Three animals were randomly selected (one from each group) and gavaged with Control or BL1.2 (both piglets from BL2.2 and BL1.2 + BL2.2 groups). Six hours after administration, piglets were culled, and digesta was collected from the stomach. Detectable amounts of active BL1.2 were measured in the stomach digesta (Table 1), demonstrating stability of BL1.2 in the stomach matrix. BL2.2 was not subjected to the same analysis, due to limitations in biomaterials.

**Table 1:** Quantification of BL1.2 in stomach content extracted from piglets

		BL1.2 quantity detected in stomach extract using Octet				
		1	2	3	Mean	Standard error of mean (SEM)
**nM BL1.2**	**Piglet (no product)**	ND	ND	13.7	–	–
	**Piglet 1 (BL1.2)**	376	383	284	347.67	31.90
	**Piglet 2 (BL1.2)**	227	485	525	412.33	93.38
**µg/mL BL1.2**	**Piglet (no product)**	ND	ND	0.0382	–	–
	**Piglet 1 (BL1.2)**	10.487	10.682	7.921	9.70	0.89
	**Piglet 2 (BL1.2)**	6.331	13.527	14.643	11.50	2.61

Piglets were fed a standard Danish sow diet and received BL1.2 via gauvage. Binding affinity of BL1.2 in stomach extracts to chip-attached FaeG (F4^+^ ETEC tip adhesin) as determined using Octet measurements. Samples were measured in triplicate, and signals measured in nM was translated to µg/mL based on the molecular weight of BL1.2.

### The piglet microbiota is stabilised by bivalent V_H_H constructs

#### Faecal microbiota composition

To assess the impact of our V_H_H constructs on the piglets’ gut microbiota, collected faecal samples (*N* = 305) throughout the in vivo study for high-throughput (Illumina) sequencing of the V3-V4 region of the bacterial 16S rRNA gene fragment. The analysis was performed on 265 DNA extracts (out of initially 305), resulting in a total of 4,335,732 high-quality sequences (per sample: Average number of reads: 16,361, Min. number of reads: 1008, Max. number of reads: 80,804) following quality control. The high-quality sequences were assigned to a total of 3295 amplicon sequence variants (ASVs) that are taxonomically distributed as presented in Supplementary Figure 4A.

**Fig. 5:**
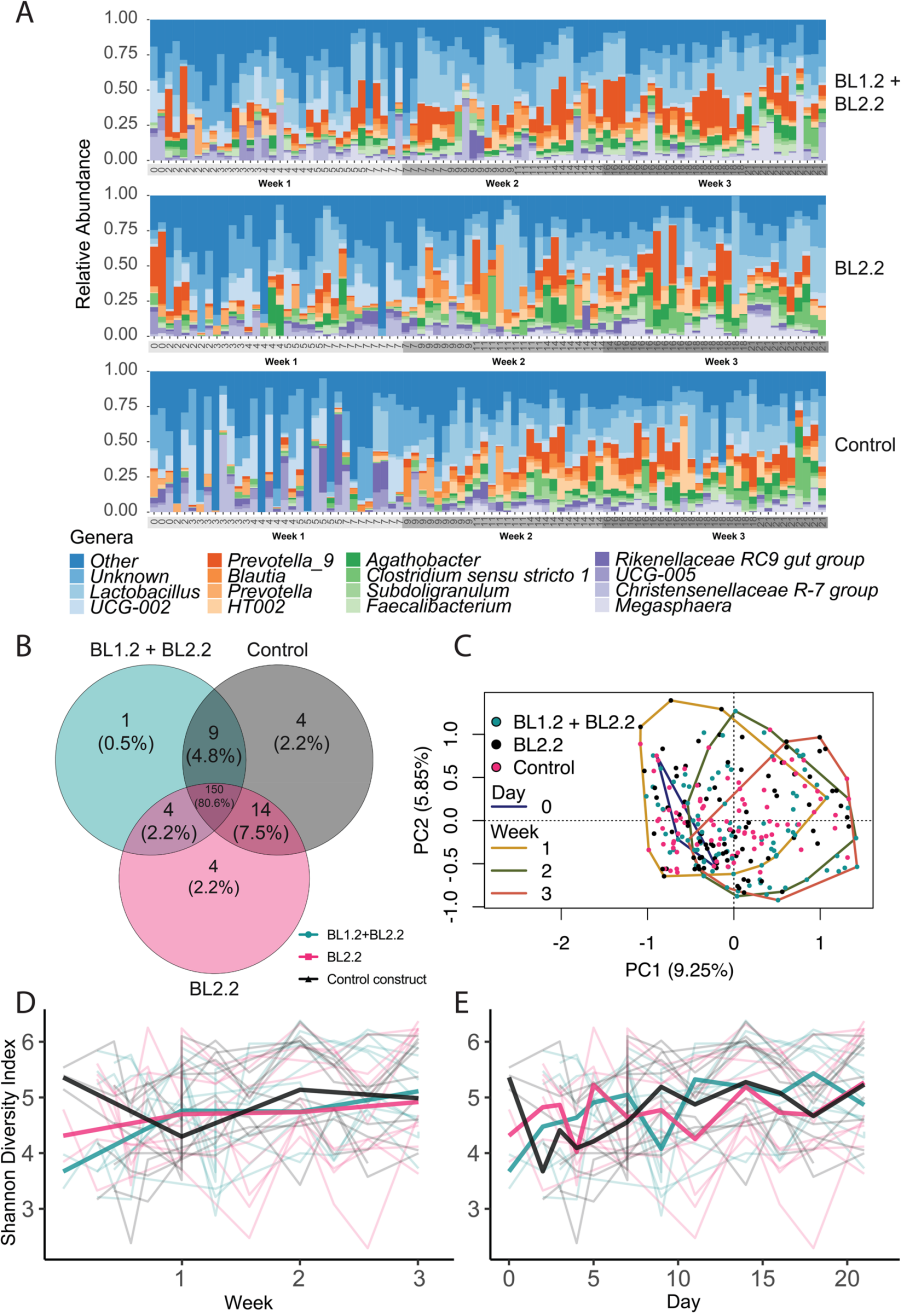
Overall temporal changes in the faecal microbiota upon ETEC challenge and supplementation with V_H_H constructs from postweaning day 0 to 21. **A** Genus-level composition across piglet groups. 16 most abundant genera depicted, with remaining collapsed as “other”. Each bar represents individual piglets at the given sampling time point (ordered chronologically). **B** Venn diagram of genus-level composition across piglet groups (circle size does not correspond to numerical values). **C** PCA plot of beta diversity of piglet faecal microbiota, based on Euclidean distances of CLR-transformed counts (ASVs). Temporal shifts in microbiota composition are evident as the analysis shows a progression across weeks, with convex hulls representing each week’s aggregate data. However, within each group (indicated by distinct colors: cyan for BL1.2 + BL2.2, black for BL2.2, and magenta for Control), the composition remains consistent, with no notable divergence between the groups over time. The background points display individual Shannon diversity values per piglet, while the bold lines chart the weekly average diversity within each group. **D** and daily averaged diversity (**E**). Visualisation of taxonomic compositions were generated from raw ASV counts as a proportion of total sample counts (relative abundance). Alpha diversity was calculated based on the Shannon index, a robust estimation of both species richness and evenness. Shannon diversity was explored overall, across each group, and between each group at weekly intervals. Holm–Bonferroni correction was applied for all multiple comparisons. See also Supplementary Figs. 4–7, and 9.

Faecal microbiota composition across the cohort was largely dominated by two phyla, encompassing ~90% of all ASVs, i.e. *Firmicutes* and *Bacteroidales* (Supplementary Fig. 4B). The most prominent phylum, *Firmicutes* (58.91%), largely consisted of members from the *Clostridiales* order (44.52%). Group-level taxonomic composition plots (Fig. 5A) provided early indications of putative genera level differences within week 1. Within week 1, on average, larger populations of *Lactobacillus, Prevotella, Prevotella 9, Blautia, HT002, Megasphaera*, and Subdoligranulum were observed in piglets which received either BL2.2 + BL1.2 or BL1.2, compared to controls (see Supplementary Table 1). However, only differences in *Prevotella 9* and *Lactobacillus* populations were later found to be significant. Several genera were found to be unique across all time points amongst piglet groups (Fig. 5B): *Arcobacter, Anaeroplasma, Enterobacter*, and *Brachyspira* were found only in control pigs, whilst *Lachnospiraceae* NC2004, *Acetanaerobacterium, Parasutterella*, and *Victivallis* were only present within groups receiving bivalent V_H_H constructs. As expected, when comparing the temporal dynamics of the *Escherichia-Shigella* genus, to which ETEC belongs, across piglet groups, it peaked within week 1 (after challenge) and gradually declined (Supplementary Figs. 5, 6) with no recorded statistically significant differences between groups. A total absence of ASVs within the *Escherichia-Shigella* genus was observed in two piglets (piglets 7 and 8), both of which received BL2.2 + BL1.2 (Supplementary Figure 6).

#### Provision of the constructs did not significantly influence bacterial diversity

Overall changes in the beta diversity among sampling weeks were found (PERMANOVA; R2 = 0.061, *F*_3,264_ = 5.67, *P* = 0.001, Fig. 5C, Supplementary Table 2) and equal dispersion was true across groups (betadispr, F_3,261_ = 1.35, *P* = 0.26). We identified no significant differences in beta diversity between the treatment groups overall, nor at individual weekly timepoints (Supplementary Figure 7A).

Alpha diversity, estimated by the Shannon index, proved highly variable across piglets. Weekly and daily averaged Shannon diversity levels fluctuated across all groups (Fig. 5D, E and Supplementary Figure 7B). For piglets receiving BL1.2 + BL2.2 or BL2.2, a net gain in average weekly Shannon diversity was 1.19 and 0.96, respectively. Both interventions exhibited similar temporal patterns with no significant differences in alpha diversity. Control piglets had a net loss in Shannon diversity (−0.13).

#### Lactobacillus and Prevotella 9 were significantly more abundant in piglets receiving BL1.2 + BL2.2

**Fig. 6:**
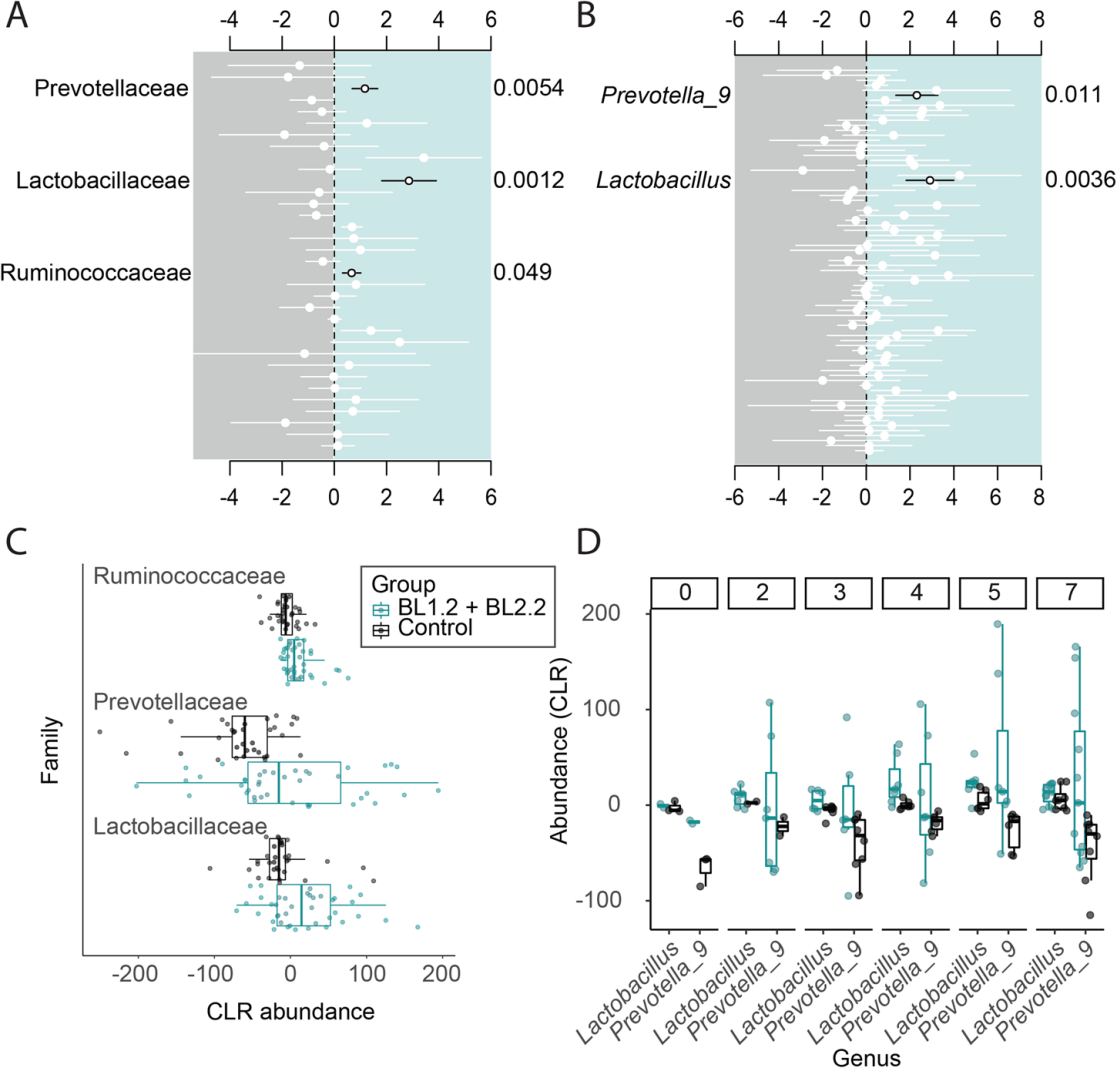
Differentially abundant taxa within week 1 for piglets receiving BL1.2 + BL2.2 vs. V_H_H controls. Differentially abundant taxa at the family level with only significant taxa annotated (**A**) and genus level (**B**). Negative binomial regression coefficients for significantly different taxa in BL1.2 + BL2.2 (blue) group, compared to control group (grey). Circles represent coefficients, with the standard error shown as bars. Adjusted *P*-values (Holm–Bonferroni) are shown on the right-hand side of the plots. Only significantly different genera are labelled (black). CLR abundances of differentially abundant families overall (**C**) and changes in genera during week 1 (**D**). See also Supplementary Fig. 8. Centre line corresponds to mean, bounds of box to lower and upper quartile and whiskers correspond to min and max. Error bars represent SD.

Differential abundance analysis of all taxa identified significant differences in family and genera level abundances at weekly intervals, with the majority of differences observed within week 1 (Fig. 6A, B). Piglets who received BL1.2 + BL2.2 had a greater abundance of *Prevotellaceae* (Estimate: 1.12 ± 0.25, q = 0.0054)*, Lactobacillaceae* (Estimate: 2.86 ± 0.52, *q* = 0.0012), and *Ruminococcaceae* (Estimate: 0.66 ± 0.18, *q* = 0.049) families compared to control piglets. The increased abundance was largely attributable to *Lactobacillus* (Estimate: 2.91 + 0.55, *q* = 0.0036) and *Prevotella 9* (Estimate: 2.31 + 0.48, *q* = 0.011) genera. For piglets who received BL2.2 only, greater levels of the *Lachnospiraceae* (Estimate = 0.94 ± 0.25, *q* = 0.043) family were detected compared to the control group (Supplementary Fig. 8). Groups receiving bivalent V_H_H constructs only differed by their level of *Rikenellaceae* family, which was greater in piglets receiving BL2.2, compared to those receiving BL1.2 + BL2.2 (Supplementary Fig. 8).

## Discussion

In this study, the utility of two orally administered bivalent V_H_H constructs as feed additives to reduce PWD in piglets, was explored in vitro and in vivo. The bivalent V_H_H constructs were demonstrated to bind and block the key virulence factors, F4^+^ fimbriae and LT, of different porcine ETEC strains in vitro without eliciting off-target effects or affecting bacterial growth rate. Interestingly, it was observed that the bivalent V_H_H construct binding F4^+^ fimbriae efficiently crosslinks bacteria to form aggregates, also during growth (Fig. 2B). This demonstrates how the proteins can have the same functionality as naturally occurring sIgA molecules, which also agglutinate, and thereby crosslink, pathogens to protect against their colonisation^21^. Furthermore, by analysing the gut microbiota composition in the piglets, it appeared that the bivalent V_H_H constructs resulted in early enrichment of certain bacterial families (*Lactobacillaceae*, *Prevotellaceae* and *Ruminococcaceae*) within week 1 post-treatment that presented later in challenged control piglets, thereby pointing to an earlier transition toward a more mature microbiota profile. In support of this, it was observed that piglets receiving BL1.2 + BL2.2 consumed 2.42 kg (+38%) and weighed 2.34 kg (+19%) more than control piglets, while piglets receiving BL2.2 alone consumed 1.37 kg (+30%) and weighed 2.29 kg (+18%) more than control piglets. While these differences in growth parameters did not reach statistical significance, likely due to limited sample sizes, they still mark substantial trends, indicating that both bivalent V_H_H constructs may benefit piglet gut health and nutrition status. Viewing the data generated in combination with the high stability of V_H_H constructs, both in relation to thermal stability with a T_m_ of 74 and 54 °C, respectively for BL1.2 and BL2.2 (Supplementary Fig. 1), and as demonstrated by retained binding activity and structure even 6 h after ingestion in this study, as well as previous studies, the findings presented here highlight the applicability of bivalent V_H_H constructs as feed additives. Consequently, we expect that the investigated bivalent V_H_H constructs are thus suitable for oral administration^19,22^ in feed or water to maintain health and support the improved growth performance of piglets.

The improvements in growth variables are notable since feed intake is typically reduced after weaning and strongly correlates with the risk of developing PWD^23^. Furthermore, underfeeding is known to lead to intestinal inflammation and adversely affects both villous height and crypt depth^24^. In turn, this presents optimal conditions for the colonisation of ETEC and allows toxins and bacteria to cross the epithelium as a result of the inflammation^25^. To address this health challenge, the antibiotic colistin has been used extensively in commercial pig production to treat PWD and promote animal growth. However, despite the known efficacy of colistin, inconclusive trial results from previous colistin studies highlight the difficulty in documenting productivity effects in piglet studies^26,27^. In trials concerning probiotic products, effects have also been difficult to document, exemplified by studies of the two different probiotics, i.e., EBS (*Enterococcus faecium* DSM 7134 + *Bacillus subtilis* AS1.836 + *Saccharomyces cerevisiae* ATCC 28338) and EBL (*Enterococcus faecium* DSM 7134 + *Bacillus subtilis* AS1.836 + *Lactobacillus paracasei* L9)^28^. Similar results were seen from a study of the use of a commercial blend of salts of medium-chain fatty acids distilled from coconut oil (Dicosan)^29^, as was the case for the use of synbiotics containing a combination of *Bifidobacterium longum* subsp. *infantis* CECT 7210 and oligofructose-enriched inulin^30^. Finally, another study utilising natural pig plasma antibodies as feed additives also show little effect, yet here an effect is reported on ETEC proliferation^31^. In comparison with these previous reports, the combination of the two bivalent V_H_H constructs, BL1.2 + BL2.2 also warrant further investigation as the improvement in productivity is not significant, yet the strong signal on reduction of ETEC proliferation and gut microbiota compositions seems promising. These results can be compared to previous work on a more complex V_H_H construct, simulating a secretory IgA molecule. In Vikram et al. [32], this IgA-like construct was shown to reduce ETEC proliferation (and hence shedding), in a similar way to our findings. While it is difficult to directly compare the generated data across the referred studies, as challenge models are different and the variance in piglet studies is very large, they do support the concept of using V_H_H constructs binding to ETEC virulence factors to sustain health in weaning piglets.

Prior studies investigating the impact of an ETEC challenge on the porcine gut microbiota have not detected major changes to gut microbial communities^33^. Yet, microbiota composition has a link to performance and productivity in piglets^34–36^. In the current study, we did not identify significant changes in alpha and beta diversity measures between the groups. However, we study across our three study groups. Indeed, in the BL1.1 + BL2.2 piglets, we found a trend that the overall alpha diversity increased across week 1, sustaining a net gain throughout the study. In contrast, control piglets experienced a temporary drop in diversity in week 1, as was earlier reported^33,37–40^, with a net loss overall. As such, there is the possibility that the bivalent V_H_H constructs may mitigate early diversity loss in piglets with ETEC-associated PWD. With the limited dataset available the findings warrant further investigation but represent an interesting trend.

We identified an early significant enrichment of three bacterial families (*Lactobacillaceae*, *Prevotellaceae* and *Ruminococcaceae*) that was represented by two genera (*Prevotella* and *Lactobacillus)* in piglets that received BL1.1 + BL2.2 in week 1, compared to those in challenged control piglets. *Prevotella* sp. produce enzymes, including xylanases and mannanases, that break down plant cell walls and enable the host to digest complex carbohydrates^41^. As such, flourishing *Prevotella* populations have been associated with increased body weight, ADG, and even feed intake in piglets^42–44^, correlating with the possible productivity increases described here. *Prevotella* spp. have also previously been negatively correlated with ETEC colonisation and diarrhoea incidence, suggesting that these bacteria may help fortify gut health in piglets^38^. Importantly, expanded *Prevotella*^45^ populations are characteristic for piglets that have transitioned to a solid diet. Whilst we lack sufficient functional data to definitively conclude that these are the mechanisms underlying our observed changes, these prior findings present a plausible hypothesis. Based on this prior knowledge, they further suggest that piglets who received BL1.2 + BL2.2 transition faster to a mature microbiota than control piglets after ETEC challenge. We speculate that the mode of action of the V_H_H constructs may be to limit the proliferation of certain taxa and promoting the expansion of other taxa. However, this will need to be proven in additional studies. Similarly, whilst non-conclusive, a plethora of studies link *Lactobacillus* spp. in the microbiota with host health; having been associated with decreased inflammatory markers, protection against enteric pathogens, and increased feed efficiency in pigs^46–48^. Both *Prevotella* and *Lactobacillus* in the pre-weaning microbiota are also identified as predictors of “healthy” (non-diarrhoeal) piglets in the weaning transition, highlighting their importance in the early gut microbiota^38^.

The economic impact of PWD, seen first and foremost as low productivity, in industrial pig production is extensive. To a farmer, improving the feed-conversion ratio by 1% conservatively saves 30 EUR/ton of feed, and this does not account for the savings related to higher animal health status and the derived lower antibiotic use. Feed for weaning piglets is the most expensive pig feed, as it includes a range of feed additives to support healthy weaning and maintain productivity. With the current rising prices of raw materials, feed prices are increasing, and optimisation of productivity is essential to ensure a financially and environmentally sustainable production system. As such, it is important to bring forward new effectful feed additives in an industry where profit margins are low, and the current use of antibiotics and medicinal zinc is unsustainable. Further, while growth promoters are still applied in some of the world’s largest pig producing regions, such as the US and China, bans and increasing restrictions leave farmers without good management tools against PWD to sustain a healthy pig production. The presented data cannot be directly translated into the commercial setting, as they are generated in a challenge model.

While this study demonstrates the utility of using the two bivalent V_H_H constructs, BL1.2 and BL2.2, to protect piglets against ETEC, some limitations remain. Similar to our previous study^19^, only a single strain was employed in the in vivo challenge trial, and other strain variants could theoretically behave differently, although the in vitro evaluation of the properties of the bivalent V_H_H constructs indicates that binding is preserved across several other ETEC strains. A known limitation of in vivo challenge studies in piglets is the difficulty in faecal sample collection, especially when piglets are small, as sample material is very limited. Further, this can be impacted by the differential feed intake that especially occurs during the first weeks after weaning. A substantial amount of variation existed between piglets across many variables, which further complicated the identification of significant differences, given the relatively small cohort size of 30 animals. Furthermore, due to ethical concerns and to enable monitoring of the piglets throughout the natural ETEC infection cycle, the ETEC challenge dose was kept low. This may have implied that the differences between ETEC-challenged piglets receiving product or no product remained modest. Additionally, to ensure adequate intake of the bivalent V_H_H constructs, the product was administered by oral gavage to the piglets, rather than the piglets being able to consume product *ad libitum* via their feed. This implies that the findings of this study may not reflect the effects of product use in a real-life industrial setting. Finally, the original BL1.2 construct was not included alone in this study, but only in combination with BL2.2. Thus, it is not possible to deduce the direct impact of each bivalent V_H_H construct on piglet health.

In this study, we showed that feeding with two bivalent V_H_H constructs reduces faecal shedding of ETEC bacteria associated with PWD by targeting the key virulence factors, F4^+^ fimbriae and LT, of porcine ETEC. In turn, the bivalent V_H_H constructs appear to facilitate earlier enrichment of certain gut bacterial families and genera upon ETEC challenge during weaning. Furthermore, a trend was observed that the two bivalent V_H_H constructs, BL1.2 and BL2.2, impacted several key growth parameters after 21 days compared to control piglets. In contrast to antibiotic and medicinal zinc treatments, which are currently in use in the industrial setting in all territories except EU where it was banned as of June 2022, it was further shown that the bivalent V_H_H constructs do not affect bacterial growth rate in vitro, even when aggregates are formed, and are highly specific against pathogenic porcine ETEC strains. Combined, these data suggest that the two bivalent V_H_H constructs, BL1.2 and BL2.2, may find utility in industrial pig production as feed additives with synergistic effects for tackling ETEC and reducing the risk of PWD in piglet populations.

## Methods

### Resource availability

#### Lead contact

Further information and requests for resources and reagents should be directed to and will be fulfilled by the lead contact Sandra Wingaard Thrane (swt@bactolife.com)

### Materials availability

This study did not generate new unique reagents.

### Experimental model and subject details

#### ETEC strains

The ETEC strain O149:F4 9910045-1 (AUF4) employed during the challenge study was collected by the Danish Veterinary Institute (Copenhagen, Denmark)^7^. We used the same F4+ strain in our previous study^19^ where we deposited its entire genome sequence to GenBank under accession number JAKLOV000000000. In addition to AUF4, we used a range of other ETEC strains collected by SEGES (Aarhus, Denmark) detailed in Table 2 for microbial growth attachment and toxin production assays.

**Table 2:** ETEC strains received by the courtesy of the SEGES Pig Research centre, Denmark

	Virulence genes (colony PCR)							
Animal ID	F4	F18	ST1	ST2	LT	VT2e	Pathology	Piglet age/weight
116387	X			X	X		Catarrhal-haemorrhagic enteritis	Ca. 1 week. Ca. 2 kg
116355	X			X	X		Faeces	N/A
116179	X		X	X	X		Catarrhal-haemorrhagic enteritis	Newly weaned. Ca. 5 kg
116130	X			X	X		Faeces	Ca. 30 kg
116026	X		X	X	X		Catarrhal-haemorrhagic enteritis	2 weeks after weaning. 9 kg
116368		X	X				Faeces	Newly weaned (4 kg)
116358		X			X		Faeces	N/A
116343		X	X	X		X	Edema in colon	9 weeks
116154		X					Faeces	N/A
116086		X		X			Faeces	Newly weaned

These strains were isolated from samples from newly deceased piglets suffering from post-weaning diarrhoea.

The isolates were routinely grown overnight (ON) in lysogeny broth (LB) with shaking (180 RPM) at 37 °C, unless stated otherwise.

#### *E. coli* BL21 (DE3)

*E. coli* BL21 (DE3) was routinely used for protein expression. Day 1: ON culture using cell scrape from glycerol stock into 5 mL LB media + 5 µL kanamycin (50 µg/mL) was grown at 37 °C, 220 RPM ON in shaking incubator. Day 2: 1 L of autoinduction media (ZY medium (10 g N-Z Amine AS, 5 g Yeast extract), 2 mL 1 M MgSO_4_, 20 mL 50 × 5052 (25% (w/v) glycerol, 2.5% (w/v) glucose, 10% (w/v) α-lactose), 20 mL 50X M (1.25 M KH_2_PO_4,_ 2.5 M NH_4_Cl, 0.25 M Na_2_SO_4_)_,_ 200 µL1000X Metals mix (50 mM FeCl_3_*6H_2_O, 20 mM CaCl_2_, 10 mM MnCl_2_*4H_2_O, 10 mM ZnSO_4_*7H_2_O, 2 mM CoCl_2_*6H_2_O, 2 mM CuCl_2_*2H_2_O, 2 mM NiCl_2_*6H_2_O, 2 mM Na_2_MoO_4_*2H_2_O, 2 mM Na_2_SeO_3_*5H_2_O, 2 mM H_3_BO_3_)) (supplemented 1 mL 50 mg/mL kanamycin) + 500 mL ON culture was placed in an incubator for 2 h at 37 °C, 200 RPM. Next, the culture was incubated at 25 °C for at least 18 h at 200 RPM shaking incubation. Further details can be found in our prior publication^19^.

#### Bivalent V_H_H constructs BL1.2 and BL2.2

Construct BL1.2 had already been designed and validated in our prior study^19^, with BL2.2 being constructed in a similar fashion for this study.

### Method details

#### Protein expression and purification

Expression and purification of recombinant proteins was performed as described in refs. ^19,49^. For the in vivo challenge study, untagged BL1.2 (27.92 kDa) and BL2.2 (28.04 kDa) constructs were produced at Novozymes laboratories (Bagsværd, Denmark). The test product was produced via microbial fermentation with secretory expression, after which the biomass was filtered, and the final test article was delivered as a frozen supernatant containing the protein product for the challenge trial. The constructs were confirmed for binding activity before being used for the trial.

#### Protein thermal shift assay

To study the thermal stability of BL1.2 and BL2.2 bivalent V_H_H constructs, Protein Thermal Shift™ assays were performed using Protein Thermal Shift Dye kit (4461146 Applied Biosystems™) and QuantStudio™ 6 pro real-time PCR instrument (Applied Biosystems™). The melting point determination was carried out by Protein Thermal Shift™ Software (Applied Biosystems™). The final assay volume was 20 µL and contained PBS buffer (pH 7.2), Protein Thermal Shift Dye (3X) and 0.5 mg/mL purified BL1.2 or BL2.2. A non-protein control was also performed. A continuously increased temperature range from 25 to 99 °C was scanned in a ramp increment of 0.05 °C per second. Scans were run in quadruplicates. The unfolding temperatures (Boltzmann T_m_) of bivalent V_H_H constructs were calculated from the inflection point of the melt curves by Protein Thermal Shift™ Software (Applied Biosystems™).

#### ETEC growth experiment

A pre-culture of *E. coli* O149:F4 9910045-1 (AUF4) denoted as ETEC F4 (Genbank: JAKLOV000000000)^7^ cells were grown over night in LB media (Sodium chloride: 27788.366 VWR, Yeast extract: 8013-01-2 Millipore, Tryptone: 95039 Millipore) at 37 °C, with shaking at 220 RPM. 50 µL culture was transferred to 500 mL fresh LB media. Samples were taken to follow colony forming units per mL (CFU/mL) and for acid elute binding assay. The 500 mL culture was divided into five flasks: one containing only ETEC F4^+^LT^+^ cells, one with 1 mg of BL1.2, one with 1 mg of BL2.2, one with both 1 mg of BL1.2 and 1 mg of BL2.2, and a final flask with 2 mg control V_H_H construct. The five flasks were incubated for 10 h at 37 °C, with agitation at 150 RPM. Five samples (technical replicates) were taken from each flask every second hour (2, 4, 6, 8, and 10) to follow CFU/mL and for acid elution binding assay. Microscopy pictures were taken at 6, 8 and 10 h after incubation using EVOS M5000 Imaging system (Invitrogen) with EVOS™ 100X/1.28 oil objective. 5 µL sample was dropped onto the object glass, covered with coverslip, and placed upside down on the mechanical stage. Vortexed samples were shaken for 10 sec at 3200 RPM with Vortex-Genie 2 (Scientific Industries, Inc.).

#### Colony forming units

To follow colony forming units per mL (CFU/mL), samples from the growth experiment were plated at dilutions ranging from 10^−3^ to 10^−7^ on LB agar plates and incubated at 37 °C ON. Colonies were counted manually. All numbers were log-transformed and means of the technical replicates at the different sampling points were statistically compared using ANOVA- test (Two-factor without replication)^50^ in excel using Data Analysis tool.

#### Acid elution binding assay

To follow binding of product to the ETEC F4^+^LT^+^ cells, samples from the growth experiment for acid elution binding assay were centrifuged at 10,000 g for 4 min, and a sample from the supernatant was taken for SDS-PAGE analysis. The pelleted cells were washed three times in 1xPBS, and bound V_H_H constructs were eluted by incubation with 0.1 M citric acid for 10 min. A sample of the eluent was taken for SDS-PAGE analysis^51^.

#### SDS-PAGE

All samples for SDS-PAGE were mixed with 4xLDS buffer (Genscript Biotech, Piscataway, NJ, USA) containing 50 mM dithiothreitol (DTT0029). Samples were boiled and loaded on NuPage 4–12% Bis-Tris protein gels (Invitrogen) together with a PageRuler prestained protein ladder (ThermoFisher scientific, Foster City, USA). The gels were run in NuPage MES SDS running buffer (Invitrogen) at 90 V for 15 min followed by 150 V for 45 min. Gels were stained using Coomassie Brilliant Blue R-250 (ThermoFisher scientific, Foster City, USA).

#### ELISA experiments

All ELISA assays were performed on Maxisorp plates (Nunc, ThermoFisher scientific, Foster City, USA). Binding analyses were performed at least in triplicates and as described in detail below.

#### Binding of construct BL2.2 to toxin-containing supernatant

For BL2.2 binding analyses, ETEC strains from Table 1 were grown in LB medium ON to late exponential phase. Cells were pelleted by centrifugation (20,000 *g* at 4 °C for 10 min) and LT-containing supernatants were normalised to an absorbance at 280 nM of 1 mg/mL (1 A/cm = 1 mg/mL) with PBS in fresh 1.5 mL reaction tubes. Maxisorp plates were coated with 100 µL/well BL2.2 (2 µg/mL in PBS) at 4 °C ON, washed 3 times with 300 µL 0.1% Tween in PBS (PBST) and blocked with 3% skimmed milk powder in PBS (M-PBS) for 1 h at room temperature. Next, plates were washed as before, incubated with 150 µL/well ETEC growth culture supernatants (1 h at room temperature), washed again and incubated with 150 µL/well FLAG-tagged BL2.2 (1 h at room temperature). Bound FLAG-tagged BL2.2 was detected using a mouse monoclonal anti-FLAG M2-Peroxidase (HRP) antibody (Sigma, diluted 1:20,000) in 3% M-PBS. Plates were developed by incubating with 3,3′,5,5′-tetramethylbenzidine–peroxide (TMB) solution, and the reaction was stopped with 2 M H_3_PO_4_. Absorbance was measured at 450 nm.

#### Binding of construct BL1.2 to adhesins from ETEC collection

Maxisorp plates were coated with heat-inactivated bacteria from the different SEGES strains diluted to an OD_600_ of 0.1 in 1xPBS. The plate was washed three times with 1xPBS and blocked with 3% skimmed milk powder (PanReac AppliChem) in 1xPBS (3% M-PBS). 60 µL (100 ng/mL) of BL1.2 was added and incubated for 1 h. The plate was washed three times with 0.1% Tween (Sigma-Aldrich) in 1xPBS (1xPBST) and 1xPBS. Bound protein was detected using a mouse monoclonal anti-FLAG M2-Peroxidase (HRP) antibody (Sigma-Aldrich), diluted 1:20,000 in M-PBS. The plate was washed three times with 1xPBST and 1xPBS and developed by incubating with Pierce™ TMB substrate Kit (ThermoFisher scientific, Foster City, USA), and the reaction was stopped with 1 M H_2_SO_4_. Absorbance was measured at 450 nm.

#### In vivo F4^+^LT^+^ ETEC challenge study with piglets

The in vivo piglet challenge study was carried out at Aarhus University (AU Viborg, Denmark) according to a licence obtained by the Danish Animal Experiments Inspectorate, Ministry of Food, Agriculture and Fisheries, Danish Veterinary and Food Administration (approval no. 2017-15-0201-01270) and the study design was approved by the institute prior to initiation. Animal care and housing were in accordance with Danish laws and regulations governing the humane care and use of animals in research.

Pigs (*n* = 30, initial body weight mean 7.9 kg STD 1.18 kg) from three sows (Yorkshire x Landrace x Duroc) fed a standard Danish starter diet were used in the study (Table 3), randomly distributed ensuring that litter from each sow is represented across all groups (Fig. 3). The sows were tested homozygous carriers of the dominant gene encoding intestinal ETEC F4 fimbriae receptors using competitive allele specific PCR (KASP) analysis of the Mucin 4 gene (VHL Genetics, Netherlands), as were the boars used for insemination of the sows. Thus, piglets were genetically susceptible to ETEC F4^+^LT^+^. The piglets were vaccinated against *Mycoplasma hyopneumoniae*, and the sows against parvovirus, *E. coli*, and swine erysipelas. Female and castrated males were included in the study. On day 23–26 after birth, piglets of both sexes were weaned, allocated to three experimental groups, balanced according to initial body weight, and housed in pens with two littermates (5 pens per group). Pairs of littermates were housed in the same pen (215 cm × 110 cm), with 75 cm × 110 cm slatted floor, and 140 cm × 110 cm concrete floor with floor heating and partial coverage. Pigs from the BL1.2 + BL2.2 and BL2.2 groups were house in the same room, each on either side of the aisle, and the Control was housed in a similar room. No physical contact was allowed between piglets from different pens. To avoid affecting the gastrointestinal system and experimental parameters, no bedding was allowed, but each pen was provided with a rope, which could help to satisfy the natural behaviour of the piglets. The room temperature was 25 °C the first week after weaning and then gradually reduced to 21 °C the third week. Piglets had access to creep feed (standard commercial feed including a flavor enhancer (Luctarom Advance) to support the piglets transition to solid feed, and even out variability due to non-eaters) from day 14 after birth and were fed *ad libitum* with the same mixture throughout the study period. Piglets were provided with drinking water *ad libitum*. Three experimental groups were randomised: pigs received a nonspecific control bivalent V_H_H construct (control); pigs received a bivalent V_H_H construct targeting ETEC LT2 toxin (BL2.2); pigs received a bivalent V_H_H construct targeting ETEC F4 fimbriae and LT2 toxin (BL1.2 + BL2.2). The BL2.2 and BL1.2 + BL2.2 groups received a 6 mL solution containing their respective constructs twice daily (morning and afternoon) for two weeks, starting at the weaning day. The solution contained 4.8 mg nonspecific or BL2.2 construct per dose and apple juice (at 1:10 w/w concentration), meaning that the piglets in group 1 and 2 received 9.6 mg/V_H_H/day). Group 3 (BL1.2 + BL2.2) received 12.1 mg construct per dose, similarly mixed with apple juice, yielding a total of 24.2 mg/V_H_H/day. On the first and second day after weaning, all groups received 5 mL of AUF4 ETEC F4^+^LT^+^ challenge strain (10^9^ CFU/mL). The AUF4 ETEC F4 + LT+ strain was grown aerobically in veal infusion broth at 37 °C for five hours with shaking (150 rpm) and OD600nm 0.2 normalised in 0.9% NaCl. Both the solution containing the constructs and that containing the AUF4 ETEC F4^+^LT^+^ were administered by placing a syringe connected to a polyethylene tube in the mouth of the piglets.

**Table 3:** Standard Danish starter diet, feed composition

Ingredients	%
Wheat	51.16
Barley	23.42
ViloSoy, Soybean protein	13.34
Potato protein	3.00
Fish meal	2.80
Palm fatty acid distillate	2.26
Sugar beat molasses	0.50
Calcium carbonate	0.76
Monocalcium phosphate	1.01
Sodium chloride	0.31
Lysine sulphate 98	0.53
Methionine DL98	0.12
Threonine 98	0.15
Tryptophan 99	0.05
Valine L 96,5	0.06
Vitamin premix	0.40
Ronozyme HiPhos	0.025
Luctarom Advance	0.10

Faecal samples from each piglet were collected directly from the rectum every day during the first week (including weaning day, but excluding days 1 and 6 post-weaning), and on days 9, 11, 14, 16, 18, and 21 post-weaning. Faecal score (following a 7-score scale, where score >3 was considered as diarrhoea); percentage of dry matter; ETEC F4+ counts; quantification of the gene encoding the F4 fimbriae (*faeG* gene) and of the heat-labile enterotoxin LT2 (*eltB* gene); and microbiota composition by 16S rRNA gene amplicon sequencing were conducted using the faecal samples (Fig. 3). At the conclusion of the study on day 21, all animals were euthanized.

Individual body weight was registered weekly, and feed intake was registered daily on a pen level.

ETEC in faeces were enumerated by counting haemolytic colonies after spread-plating on blood agar (Columbia blood agar medium supplemented with sheep blood, Thermo Fisher Scientific, Waltham, Massachusetts, USA) and incubating aerobically ON at 37 °C. The limits of detection were between 10^4^ and 10^6^ CFU/g faeces, depending on expected counts in the different days. ETEC F4^+^ serotyping was performed using the slide agglutination test with type-specific antisera (SSI Diagnostica A/S, Hillerød, Denmark) on five colonies per plate.

#### Quantification of BL1.2 in piglet stomach digesta

At the conclusion of the in vivo study, before the animals were culled, three piglets were selected (one from each group) and gavaged with 5 mL control or BL1.2 construct, respective to their group allocations. The piglets were not fasted, and had access to feed ad libitum, like they had during the trial, to see also the product going through the GI tract, in combination with a normal feed matrix. Each animal was sacrificed 6 h after the procedure using a captive bolt gun followed by immediate exsanguination. Stomach and intestinal content was collected in sterile containers and stored at −80 °C until further use. The samples were freeze-dried to maintain sample integrity and standardisation of samples. Samples were resuspended in sterile PBS solution (100 mg of sample in 1 mL) and centrifuged at 12,000 g for 10 min. To verify the presence of BL1.2 from purified supernatants, samples were analysed by biolayer interferometry on an Octet® Red96 instrument (Sartorius, Göttingen, Germany). NTA biosensors (Sartorius, Göttingen, Germany) were hydrated in 1xkinetics buffer (1xKB, Sartorius) and then loaded with His-tagged FaeG (5 µg/mL in 1xKB, incubation time of 300 s). Following a baseline step for 60 s in 1xKB, the biosensors were dipped in sample solutions (centrifuged at 14,000 *g* for 10 min prior to use). Analyte association was allowed to take place for 300 s. All steps were performed at 30 °C with a shake speed (flow rate) of 1000 RPM. All samples were diluted ten-fold in 1xKB and measured in duplicate. Binding rates were determined in the Octet Data Analysis software (v 12.2), using the initial slope binding rate equation (read time set at 60 s). The standard curve was generated based on 7 different concentrations of BL1.2 (2-fold dilution series starting at 100 nM, in sample matrix diluted ten-fold in 1xKB), which were analysed in duplicate. The unweighted 4PL equation was employed for fitting the standard curve to the data points. Due to the composition of the intestinal samples, readings were inconclusive, and data could only be generated for samples from stomach content.

#### Quantification and statistical analysis

Differences in pig growth performance were assessed using a linear mixed effects model. The model included treatment as a fixed effect, initial body weight was included as a covariate for ADG and ADFI, and pen and sow effects were included as random effects. Assumptions of normality and homogeneity of variance were confirmed. Satterthwaite approximation was used for denominator degrees of freedom. Results from faecal samples were analysed by a normal or lognormal linear mixed effects model with treatment, day (as categorical variable) and their interaction as fixed effects, and the random effects of pen and sow. A continuous-time autoregressive covariance structure of order 1 was used to account for correlation among measures from the same pig. Due to many samples classified as non-diarrhoeic, it was not possible to estimate diarrhoea prevalence. Therefore, arithmetic means with standard deviations of the faecal scores are presented as Supplementary Information. When there was an overall effect of diet at an alpha of P < 0.05, and the interaction between diet and day was not significant, differences between means were compared pairwise using P-values adjusted for multiple comparisons using the Holm–Bonferroni adjustment. The data are shown as Least Square Means and standard error unless otherwise stated. Figures were made in GraphPad Prism 9.5.1.1.

#### DNA extraction and quantification of virulence genes using qPCR

Quantitative polymerase chain reaction (qPCR) was used for quantification of the gene encoding the F4ac fimbriae (*faeG* gene) and the heat-labile enterotoxin LT2 (*eltB* gene) in faecal samples. Briefly, DNA extraction was performed using the NucleoSpin® 96 Stool kit (MACHEREY-NAGEL GmbH & Co. KG, Düren, Germany), following the manufacturer’s instructions for vacuum processing. Qubit 3.0 Fluorometer was used to measure the concentration of the DNA samples at room temperature. Quantitative PCR reactions were run using ViiA 7 Real-time PCR System (ThermoFisher scientific, Foster City, USA), targeting both the *faeG* and the *eltB* gene. Primers are detailed in Table 4. The reaction mix consisted of MasterMix containing 4-5 µL RealQ Plus 2x Master Mix Green (Amplicon, Denmark), 0.3 µL of the forward and reverse primer (10 pmol/µL working stock), and DEPC treated water to reach 8 µL volume per well; additionally, 2 µL of DNA template was added to the 384-well plate. Each DNA sample was measured using triplicates, and selectivity confirmed by using non-template controls (NTC). Serially diluted genomic DNA isolated from a bacterium that contained the target gene served as internal control. Heat profile used with the *faeG* primer pair consisted of a 2 min initial heating at 50 °C, a hot start step for 15 min at 95 °C, and a three-step reaction (15 s at 95 °C, 30 s at 65 °C, and 30 s at 72 °C) repeated 40 times. This was followed by a melting curve analysis. The same program was used with *eltB* primers, except for the annealing temperature, which was decreased to 60 °C.

**Table 4:** Primers used for quantitative PCR

Target gene	Primer direction	Sequence 5’ → 3’	Template size
*faeG* (F4ac fimbriae)	F	CACTGGCAATTGCTGCATCT	86 bp
	R	ACCACCGATATCGACCGAAC	
*eltB* (LT2 toxin)	F	AAGCCATTGAAAGGATGAAGGA	112 bp
	R	CTGATTGCCGCAATTGAATT	

#### 16S rRNA gene amplicon sequencing

DNA samples (same as used for qPCR) were diluted to 5 ng/µL concentration using nuclease free water. PCR was performed on a UNO96 thermocycler (VWR, Radnor, PA, US) amplifying the V3-V4 region of the 16 S rRNA gene using Illumina_16S_341F (5′ -TCGTCGGCAGCGTCAGATGTGTATAAGAGACAGCCTACGGGNGGCWGCAG) and Illumina_16S_805R (5′-GTCTCGTGGGCTCGGAGATGTGTATAAGAGACAGGACTACHVGGGTATCTAATCC) primers^52^. Reaction mixture contained 0.25 µL Phusion™ High-Fidelity DNA Polymerase (ThermoFisher Scientific, Foster City, USA), 5 µL HF buffer, 1-1 µL of forward and reverse primer (10 µM), 2 µL of template DNA, and molecular grade water to reach final volume of 25 µL. the thermal profile of the run was as follows: 1 min initiation at 95 °C; 32 cycles of 15 s at 95 °C, 15 s at 56 °C, and 30 s at 72 °C; followed by a final elongation step for 10 min at 72 °C. Verification of successful amplification was done by agarose gel electrophoresis using 4 µL PCR product. The remaining liquid was subjected to clean-up by AmpureXP magnetic beads (Beckman Coulter Inc., Brea, CA, USA), following recommendations by the producer and eluted in 20 µL nuclease free water. Indexing PCR was performed using a series of forward and reverse custom designed primers (Table 2) that are compatible with the Illumina sequencing platform. The indexing reaction contained 0.25 µL Phusion™ High-Fidelity DNA Polymerase (ThermoFisher scientific, Foster City, USA), 5 µL HF buffer, 2 µL of purified PCR product, and molecular grade water to reach final volume of 21 µL. 2.-2 µL of forward (unique for each column) and reverse (unique for each row) indexing primers (10 µM concentration) were arranged on the 96 well plate to achieve a matrix of primer combinations for each individual well. This was followed by a subsequent clean-up using AmpureXP magnetic beads. The concentration of each sample was determined by a Qubit dsDNA high-sensitivity (HS) kit. Before pooling the samples for sequencing, their concentration was equilibrated to 4 nM. Amplicon libraries were sequenced using the Illumina MiSeq Reagent Kit V3 (600 Cycles) with 300 bp paired-end reads. This resulted in a total read count of 4,335,732 after filtering (16,361 average reads per sample).

### Microbiota analysis

Before processing of reads, the primers were removed from the raw sequences using Cutadapt (v 4.1)^53^. Subsequently, the primer clipped sequences were loaded to R (v 4.1.3) and processed with the DADA2 pipeline in the R package (v 1.22.0) according to Callahan et al.^54^. Forward and reverse sequences were trimmed to 260 and 230 bp, respectively, and further 5 bp were trimmed from the left side of the sequences to remove the remaining low-quality bases at the beginning. Additional filtering included, read truncation at a quality score cut-off (truncQ = 2) and removal of reads with high expected errors (maxEE = 2 (forward read), 5 (reverse read)). Error correction, merging, and chimera removal were performed using the default parameters. Finally, taxonomy assignment was done using the Naive Bayesian Classifier and the DADA2 formatted Silva v.138.1.

All statistical analysis was performed in R (v 4.1.3). Potential contaminants were identified and removed with Decontam^55^ using a prevalence-based approach (threshold *p* = 0.5). Any remaining ASVs that were present in the negative controls and not in true samples were also removed (Supplementary Table 3). ASVs, that were not assigned to the kingdom of bacteria or were not classified lower than the kingdom level, were removed. ASVs with no more than one read were also removed^56^. Finally, samples with less than 1000 reads in total were removed, as the sequencing was not considered of sufficient depth (Supplementary Table 4). Rarefaction curves were generated to ensure that sufficient sequencing of samples was achieved (Supplementary Figure 9).

Microbiota data are inherently compositional, and ASV counts were transformed using the centred log(2)-ratio (CLR) to account for this, as recommended by Gloor et al.^57^. A principal component analysis (PCA) was performed on a Euclidean distance matrix generated from CLR transformed counts. Significant differences in beta diversity were assessed using permutation multivariate analysis of variance (PERMANOVA) in vegan^58^. Statistical approaches to handle repeated measures in beta diversity analyses are limited, and to adjust for this, we restricted permutations via the “strata” argument to individual piglets. Pairwise PERMANOVA comparisons were conducted using the pairwise Adonis package^59^. Alpha diversity was calculated at ASV level and was based on the Shannon index, a robust estimation of both richness and evenness, using the microbiome package^60^. Changes in Shannon diversity over time were assessed using the splinectomeR package^61^. splinectomeR fits a loess spline to data and assesses if trends in diversity significantly differ from a null distribution, calculated through a permutational approach. Shifts in Shannon diversity were explored overall, across each group, and between each group at weekly intervals. ETEC prevalence in faecal microbiota was assessed based on ASV populations within the *Escherichia-Shigella* genera in terms of both relative abundance and total read count. Differential abundance testing was performed using the Negative Binomial and Zero-Inflated Mixed Models (NBZIMM) package^62^. Zero inflated negative binomial models tested for differences in the abundance of taxa aggregated to family and genera levels, on a weekly basis, whilst adjusting for variation in library size, time of sampling (days) and individuals. Default parameters were kept, with the exception of the minimum proportion parameter (min. *p* = 0.2), which sets the inclusion criteria to retain taxa with a given nonzero proportion. Visualisation of taxonomic compositions was generated from raw ASV counts as a proportion of total sample counts (relative abundance). Holm–Bonferroni correction was applied for all instances of multiple comparisons.

### Reporting summary

Further information on research design is available in the Nature Research Reporting Summary linked to this article.

### Supplementary information

Supplementary Information

Reporting summary

## Data Availability

The raw data and data frames have been deposited in Mendeley Data, V1, 10.17632/5fgvkb4hyz.1.

## Code availability

The raw data and data frames have been deposited in Mendeley Data, V1, 10.17632/5fgvkb4hyz.1.
