# Supplementary material for: Protecting the piglet gut microbiota against ETEC-mediated post-weaning diarrhoea using specific binding proteins
Source: NPJ Biofilms Microbiomes. 2024 May 2;10:42. doi: 10.1038/s41522-024-00514-8 (PMC11066037; doi:10.1038/s41522-024-00514-8)
Supplement: Supplementary file 1 — Supplementary Information [file 41522_2024_514_MOESM1_ESM.pdf]

Supplementary Information

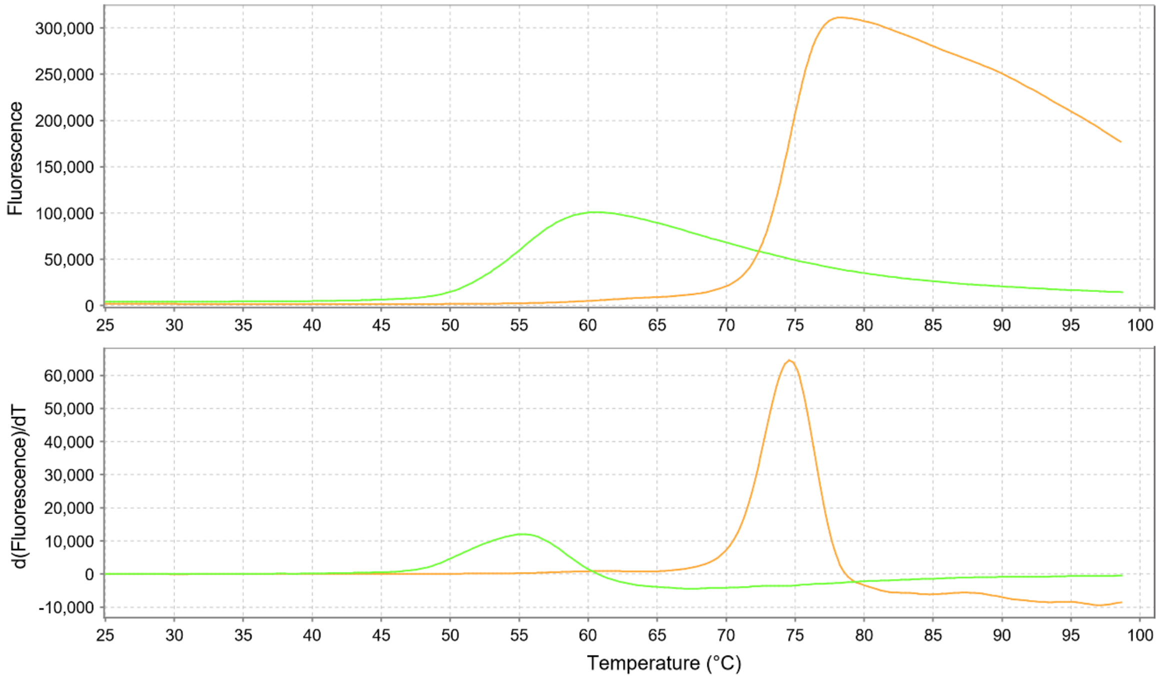

Supplementary Figure 1: Thermal denaturation profile of bivalent V_HH constructs. Based on melt curve and derivative plot of BL1.2 (orange line) and BL2.2 (green line) unfolding temperatures were calculated. Boltzmann T_m points of BL1.2 and BL2.2 are 74 °C and 54 °C.

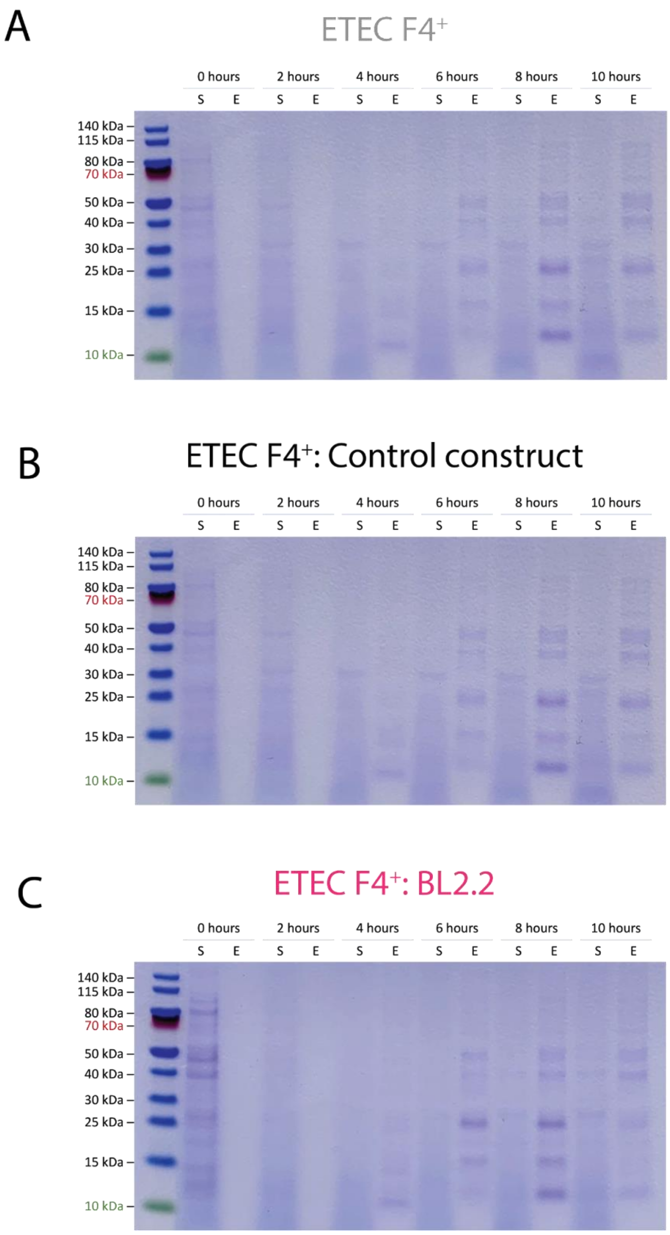

Supplementary Figure 2: Related to Figure 2. SDS-PAGE experiment indicating no construct adhesion to $F4^+LT^+$ ETEC neither by the control construct with expected migration at ~27.6 kDa (B) or by BL2.2 with expected migration at ~28 kDa (C).

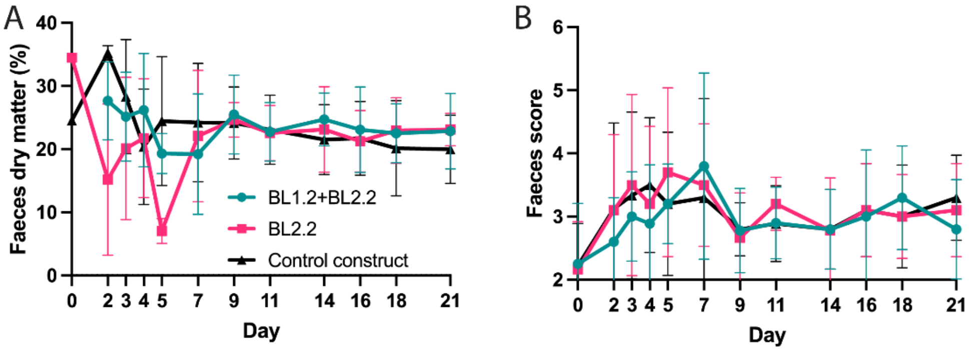

Supplementary Figure 3: Related to Figure 4. To assess the impact of the V_HH constructs, the percentage of dry matter in the piglet faeces (A) and their faeces scores (B) were monitored throughout the study. Error bars represent mean +/- SD.

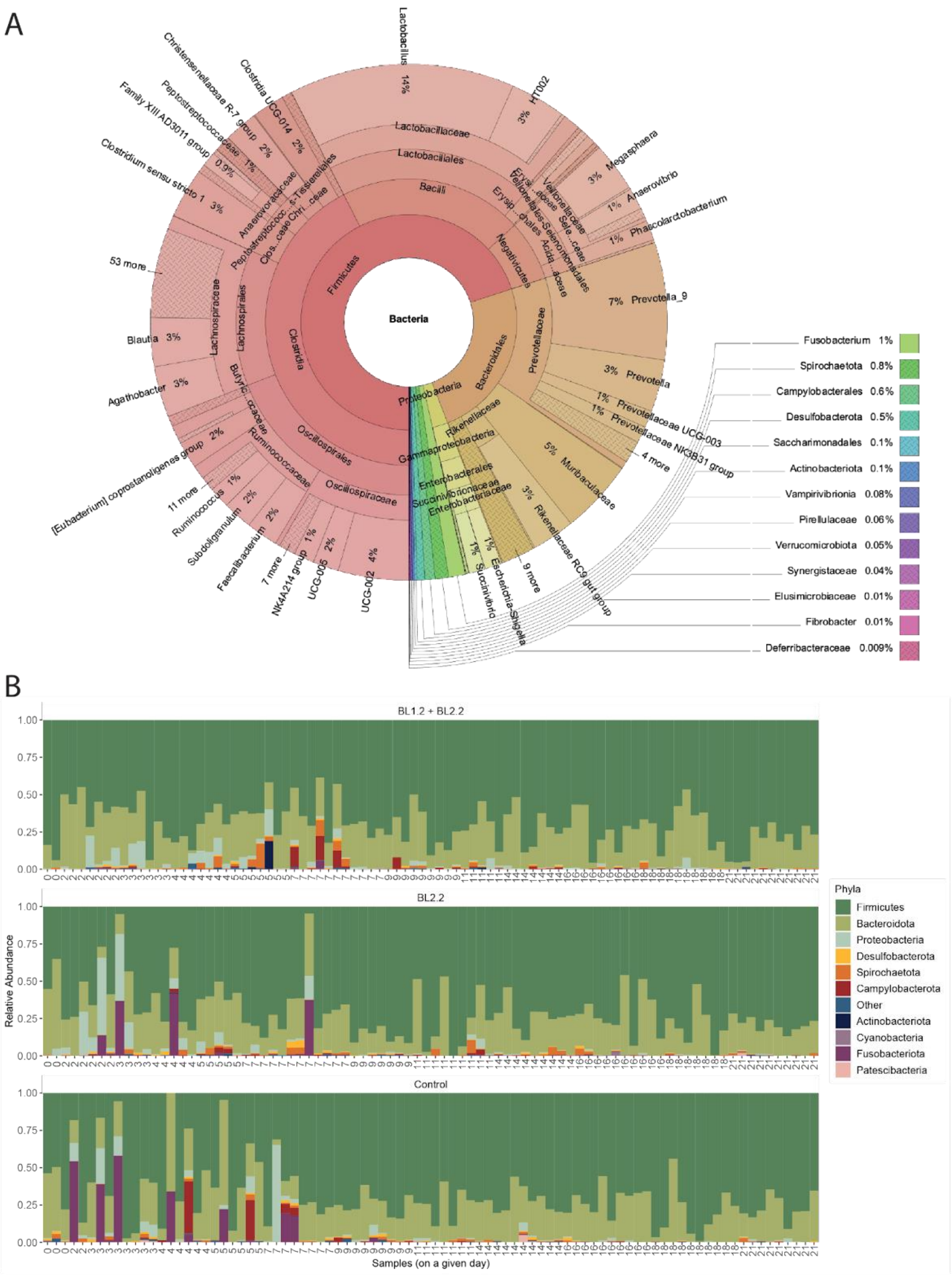

Supplementary Figure 4: Faecal microbiota composition. Related to Figure 5. (A) Krona plot (created with Kronatools) showing multi-level taxonomic composition of all faecal samples (mean) from domain to genus level. (B) Phylum-level composition across piglet groups. Ten most abundant phyla depicted, with remaining collapsed as "other". Each bar represents individual piglets at a sampling time point (ordered chronologically).

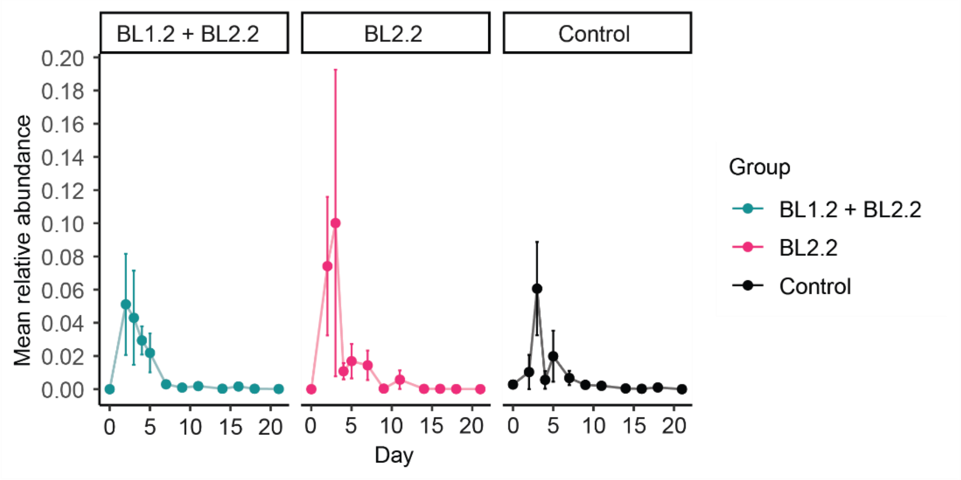

Supplementary Figure 5. Per group ETEC detection in faecal microbiota. Related to Figure 5. Abundances shown here are based on aggregated ASVs within the ‘*Escherichia-Shigella*’ genus that ETEC belongs to. Average *Escherichia-Shigella* relative abundance throughout time across the piglet groups. Data represents mean +/- SEM, and error bars represent mean +/- SD

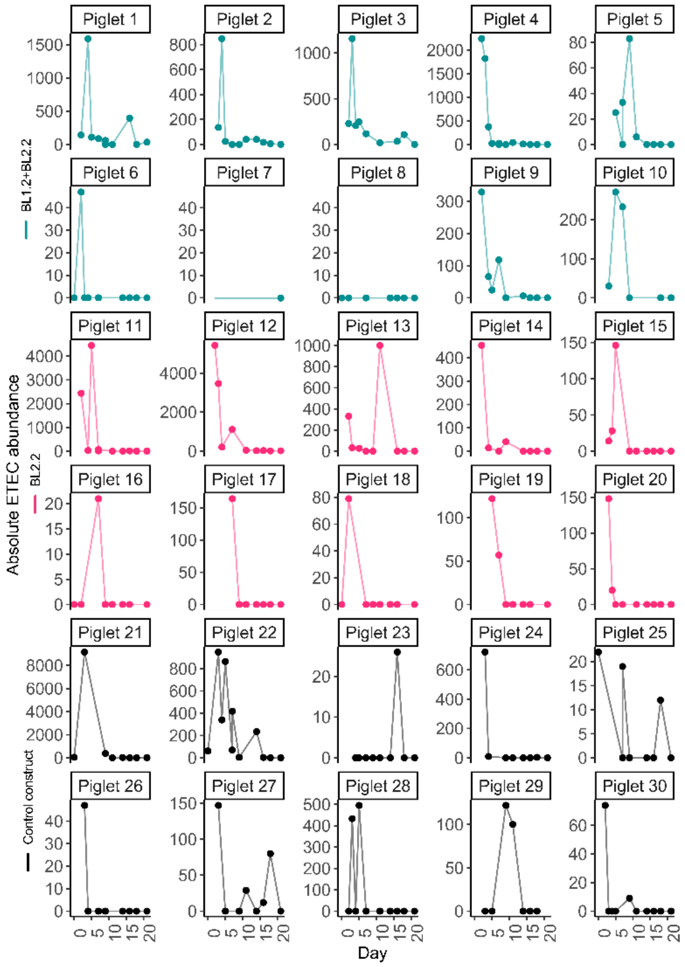

Supplementary Figure 6: ETEC detection in faecal microbiota. Related to Figure 5. Absolute abundances are shown per piglet and are based on aggregated ASVs within the '*Escherichia-Shigella*' genus that ETEC belongs to. Samples were not available across all time points for all piglets. No ASVs pertaining to *Escherichia-Shigella* were found in the faecal microbiota of piglet 7 and 8.

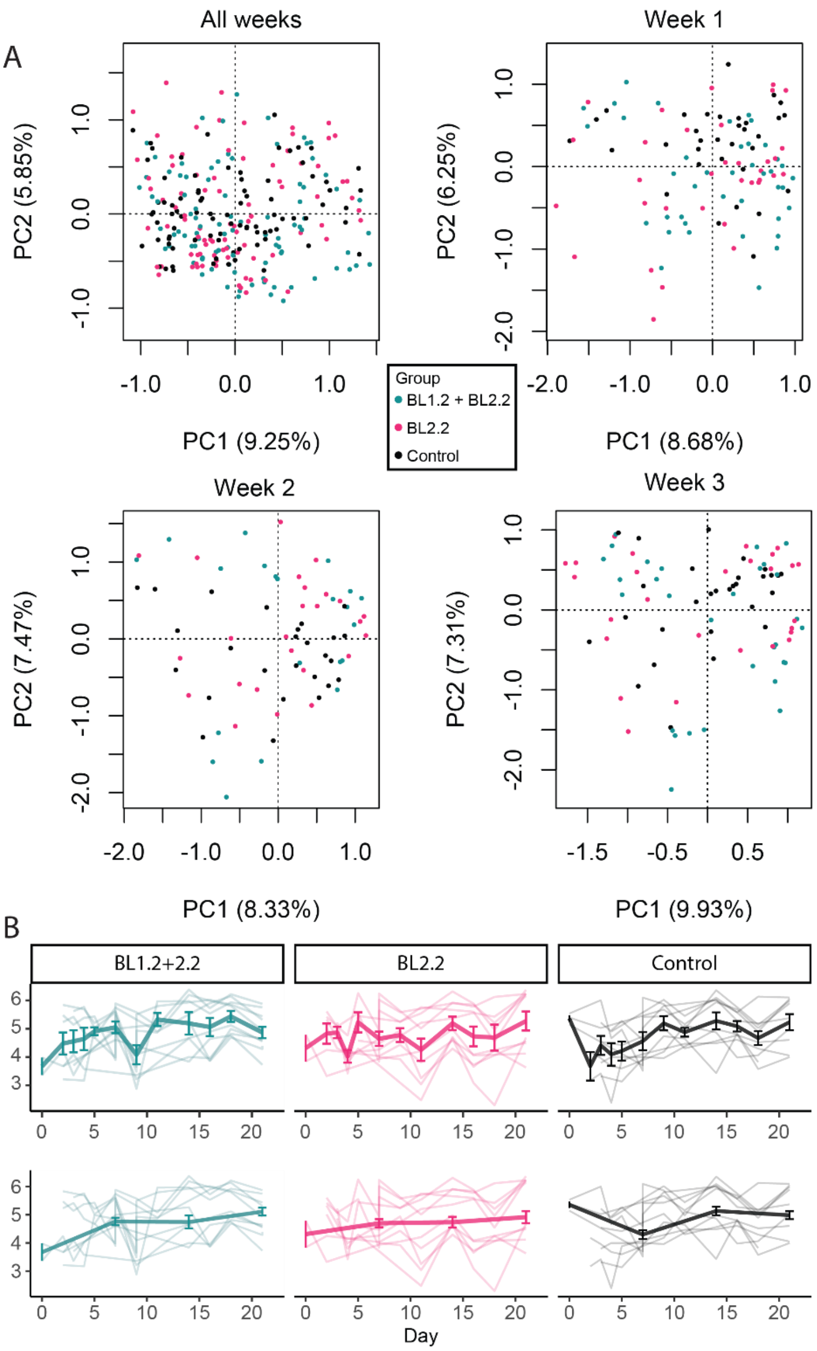

Supplementary Figure 7: Diversity of faecal microbiota. Related to Figure 5. (A) PCA plots of beta diversity of piglet faecal microbiota based on Euclidean distances of CLR transformed counts (ASVs) overall, and for each week. Colour of points correspond to treatment group. No clustering of beta diversity can be seen according to group, and no significant differences in beta diversity were detected between groups with PERMANOVA. (B) Alpha diversity (Shannon diversity index) throughout time across treatment groups. The top panel shows daily average Shannon diversity (in bold) per group with SD error bars, whilst bottom panel shows weekly averaged Shannon diversity (in bold) per group. Alpha diversity trajectories per piglet can be seen in the background of plots.

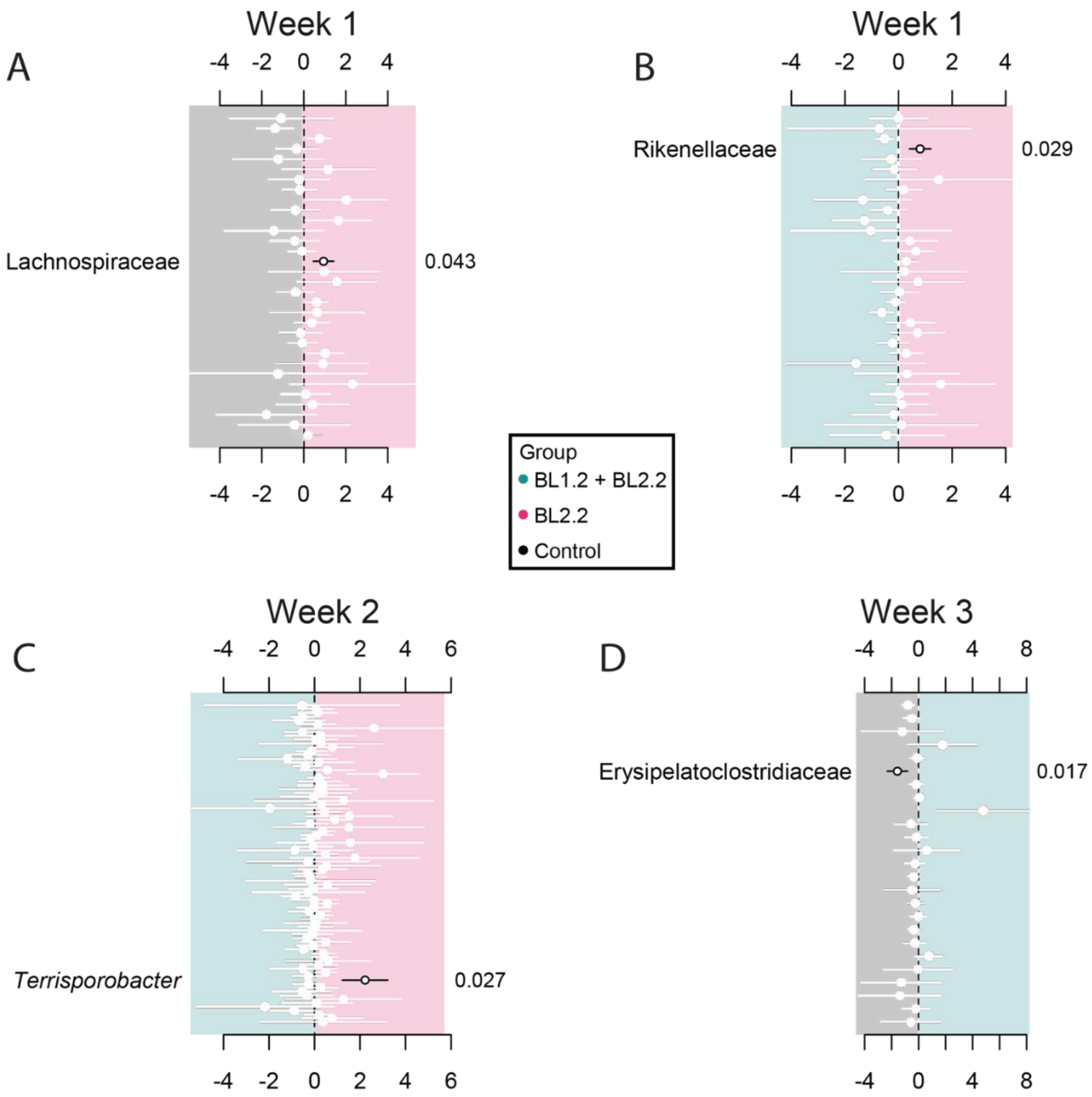

Supplementary Figure 8: Differentially abundant taxa across dietary groups. Related to Figure 6. Differentially abundant families in week 1 (A,B) between piglets who received BL2.2 relative to controls (A) and piglets who received BL2.1+BL2.2 (B). Differentially abundant genera (C,D) in week 2 between piglets who received BL2.1, relative to those which received BL2.1+BL2.2 (C). Differentially abundant genera in week 3 between piglets who received BL2.1+BL1.2 relative to controls (D). Differences between means were compared pairwise using P-values adjusted for multiple comparisons using the Holm-Bonferroni adjustment. The colours represent which significantly different taxa is associated with a group, e.g. if a taxa falls within the “pink” region, this is associated as an increase in BL2.2.

Fig S9.

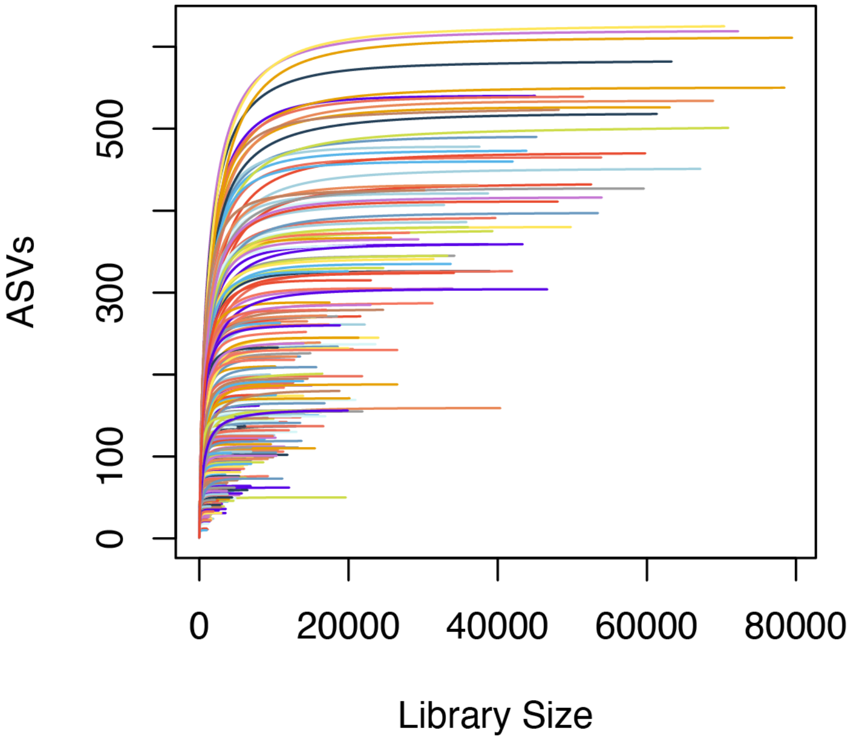

Supplementary Figure 9: Rarefaction curve showing ASV diversity of samples (post-filtering). Related to Figure 5. All samples across different library sizes represented as different colour lines show saturation of ASV diversity. Rarefaction curve generated using the rarecurve function in vegan (step = 20).

Supplementary Table 1: Summary statistics of genus-level relative abundance for week 1 across piglet groups. Top 16 genera are shown. Mean average (AV) with standard deviation, Standard error (SE) and the minimum and maximum values (RANGE) shown. Genera which were, on average, higher in piglets receiving BL2.2 + BL1.2 or BL1.2 compared to controls are highlighted in bold.

**OTU**	**BL2.2 + BL1.2**	**BL1.2**	**Control**
Agathobacter	AV: 0.021 ± 0.031,<br>SE: 0.0051,<br>RANGE: 0-0.13	AV: 0.028 ± 0.066,<br>SE: 0.012,<br>RANGE: 0-0.26	AV: 0.0029 ± 0.0093,<br>SE: 0.0017,<br>RANGE: 0-0.037
Anaerovibrio	AV: 0.0092 ± 0.011,<br>SE: 0.0019,<br>RANGE: 0-0.04	AV: 0.013 ± 0.015,<br>SE: 0.0026,<br>RANGE: 0-0.052	AV: 0.0059 ± 0.013,<br>SE: 0.0023,<br>RANGE: 0-0.065
Blautia	AV: 0.015 ± 0.016,<br>SE: 0.0025,<br>RANGE: 0-0.073	AV: 0.021 ± 0.034,<br>SE: 0.006,<br>RANGE: 0-0.16	AV: 0.0081 ± 0.013,<br>SE: 0.0023,<br>RANGE: 0-0.039
Christensenellaceae R-7 group	AV: 0.044 ± 0.086,<br>SE: 0.014,<br>RANGE: 0-0.39	AV: 0.035 ± 0.04,<br>SE: 0.007,<br>RANGE: 0-0.13	AV: 0.084 ± 0.13,<br>SE: 0.024,<br>RANGE: 0-0.55
***Clostridium sensu stricto* 1**	AV: 0.0063 ± 0.01,<br>SE: 0.0016,<br>RANGE: 0-0.053	AV: 0.017 ± 0.02,<br>SE: 0.0035,<br>RANGE: 0-0.066	AV: 0.016 ± 0.021,<br>SE: 0.0038,<br>RANGE: 0-0.078
Faecalibacterium	AV: 0.019 ± 0.021,<br>SE: 0.0034,<br>RANGE: 0-0.077	AV: 0.011 ± 0.016,<br>SE: 0.0029,<br>RANGE: 0-0.065	AV: 0.0019 ± 0.0045,<br>SE: 0.00082,<br>RANGE: 0-0.021
**HT002**	AV: 0.022 ± 0.034,<br>SE: 0.0054,<br>RANGE: 0-0.12	AV: 0.0084 ± 0.02,<br>SE: 0.0035,<br>RANGE: 0-0.072	AV: 0.0044 ± 0.011,<br>SE: 0.0019,<br>RANGE: 0-0.044
Lactobacillus	AV: 0.13 ± 0.14,<br>SE: 0.023,<br>RANGE: 0-0.45	AV: 0.062 ± 0.12,<br>SE: 0.021,<br>RANGE: 0-0.43	AV: 0.025 ± 0.074,<br>SE: 0.014,<br>RANGE: 0-0.31
Megasphaera	AV: 0.014 ± 0.026,<br>SE: 0.0042,<br>RANGE: 0-0.1	AV: 0.0011 ± 0.0041,<br>SE: 0.00073,<br>RANGE: 0-0.022	AV: 0.00024 ± 0.00062,<br>SE: 0.00011,<br>RANGE: 0-0.0026
Other	AV: 0.31 ± 0.17,	AV: 0.37 ± 0.21,	AV: 0.41 ± 0.32,

	SE: 0.028, RANGE: 0.055-0.65	SE: 0.038, RANGE: 0.041-0.94	SE: 0.059, RANGE: 0.05-1
Prevotella	AV: 0.029 ± 0.039, SE: 0.0063, RANGE: 0-0.24	AV: 0.035 ± 0.033, SE: 0.0059, RANGE: 0.0016-0.16	AV: 0.02 ± 0.028, SE: 0.005, RANGE: 0-0.091
***Prevotella* 9**	AV: 0.09 ± 0.11, SE: 0.018, RANGE: 0-0.47	AV: 0.032 ± 0.049, SE: 0.0087, RANGE: 0-0.21	AV: 0.0076 ± 0.013, SE: 0.0023, RANGE: 0-0.053
Rikenellaceae RC9 gut group	AV: 0.011 ± 0.01, SE: 0.0016, RANGE: 0-0.044	AV: 0.025 ± 0.027, SE: 0.0048, RANGE: 0-0.095	AV: 0.061 ± 0.11, SE: 0.02, RANGE: 0-0.5
Subdoligranulum	AV: 0.015 ± 0.014, SE: 0.0022, RANGE: 0-0.055	AV: 0.012 ± 0.016, SE: 0.0027, RANGE: 0-0.067	AV: 0.0044 ± 0.0067, SE: 0.0012, RANGE: 0-0.026
UCG-002	AV: 0.069 ± 0.067, SE: 0.011, RANGE: 0-0.27	AV: 0.097 ± 0.096, SE: 0.017, RANGE: 0-0.41	AV: 0.14 ± 0.14, SE: 0.026, RANGE: 0-0.42
UCG-005	AV: 0.025 ± 0.044, SE: 0.0071, RANGE: 0-0.24	AV: 0.02 ± 0.024, SE: 0.0043, RANGE: 0-0.12	AV: 0.033 ± 0.06, SE: 0.011, RANGE: 0-0.26
**Unknown**	AV: 0.17 ± 0.091, SE: 0.015, RANGE: 0.027-0.47	AV: 0.21 ± 0.1, SE: 0.018, RANGE: 0.029-0.41	AV: 0.17 ± 0.14, SE: 0.025, RANGE: 0-0.44

Supplementary Table 2. Microbial composition across weeks significantly differs. Related to Figure 5. Pairwise permanova performed on complete faecal microbial dataset across weeks (0, 1, 2, and 3), with strata set to individual pigs to adjust for repeated measures. All pairwise PERMANOVA comparisons were found to be significant. q-value represents adjustment of *p*-value for multiple comparisons by the Holm-Bonferroni approach.

Comparison	*p*-value	q-value
0 vs 1	0.003	0.006
0 vs 2	0.001	0.006
0 vs 3	0.001	0.006
1 vs 2	0.001	0.006
1 vs 3	0.001	0.006
3 vs 2	0.001	0.006

Supplementary Table 3. Removal of microbial taxa. Related to Figure 5. Taxa identified to either be contaminant species and/or only identified in the control samples.

ASV	Faecal sample prevalence (*n* = 266)	Negative control sample prevalence (*n* = 3)	Removal purpose	Taxa
ASV_1617	0	2	Decontam identified contaminant	*Cutibacterium namnetense*
ASV_1188	0	1	Only present in control samples	*Aquipuribacter hungaricus*
ASV_2291	0	1	Only present in control samples	*Cutibacterium (*uncharacterised*)*
ASV_2790	0	1	Only present in control samples	*Aquipuribacter (uncharacterised)*
ASV_3169	0	1	Only present in control samples	*Methylobacterium Methylorubrum*
ASV_5112	0	1	Only present in control samples	*Sphingomonas (uncharacterised)*
ASV_6771	0	1	Only present in control samples	*Acinetobacter (uncharacterised)*

Supplementary Table 4. Related to STAR Methods. Samples dropped from study during post-classification quality control steps (*n* = 56), due to insufficient quality. Though considerable sample loss occurred, a relatively even loss across treatment groups and timepoints was observed.

	Total samples lost	% of all corresponding samples lost
Control (*n* = 102)	14	13.73%
BL2.2 (*n* = 104)	18	17.31%
BL1.2+BL2.2 (*n* = 111)	20	18.1%
Within week 1 (*n* = 133)	26	19.56%
Within week 2 (*n* = 94)	22	23.40%
Within week 3 (*n* = 90)	4	4.44%
