# Supplementary material for: Protecting the piglet gut microbiota against ETEC-mediated post-weaning diarrhoea using specific binding proteins
Source: NPJ Biofilms Microbiomes. 2024 May 2;10:42. doi: 10.1038/s41522-024-00514-8 (PMC11066037; doi:10.1038/s41522-024-00514-8)
Supplement: Supplementary file 2 — Reporting summary [file 41522_2024_514_MOESM2_ESM.pdf]

nature portfolio

Corresponding author(s): Andreas Hougaard Laustsen, Susanne Brix, Sandra Wingaard Thrane

Last updated by author(s): Oct 23, 2023

Reporting Summary

Nature Portfolio wishes to improve the reproducibility of the work that we publish. This form provides structure for consistency and transparency in reporting. For further information on Nature Portfolio policies, see our Editorial Policies and the Editorial Policy Checklist.

Statistics

For all statistical analyses, confirm that the following items are present in the figure legend, table legend, main text, or Methods section.

n/a	Confirmed	
☐	☒	The exact sample size (*n*) for each experimental group/condition, given as a discrete number and unit of measurement
☐	☒	A statement on whether measurements were taken from distinct samples or whether the same sample was measured repeatedly
☐	☒	The statistical test(s) used AND whether they are one- or two-sided<br>*Only common tests should be described solely by name; describe more complex techniques in the Methods section.*
☐	☒	A description of all covariates tested
☐	☒	A description of any assumptions or corrections, such as tests of normality and adjustment for multiple comparisons
☐	☒	A full description of the statistical parameters including central tendency (e.g. means) or other basic estimates (e.g. regression coefficient) AND variation (e.g. standard deviation) or associated estimates of uncertainty (e.g. confidence intervals)
☐	☒	For null hypothesis testing, the test statistic (e.g. *F*, *t*, *r*) with confidence intervals, effect sizes, degrees of freedom and *P* value noted<br>*Give P values as exact values whenever suitable.*
☒	☐	For Bayesian analysis, information on the choice of priors and Markov chain Monte Carlo settings
☒	☐	For hierarchical and complex designs, identification of the appropriate level for tests and full reporting of outcomes
☒	☐	Estimates of effect sizes (e.g. Cohen's *d*, Pearson's *r*), indicating how they were calculated

Our web collection on statistics for biologists contains articles on many of the points above.

Software and code

Policy information about availability of computer code

Data collection	Data was collected and recorded in Excel (16.78)
Data analysis	Statistical analysis of growth and performance data was performed in GraphPad Prism 9.5.1. NGS reads for microbiota analysis were pre-processed using Cutadapt (v 4.1). Subsequently, the primer clipped sequences were loaded to R (v 4.1.3) and processed with the DADA2 pipeline in the R package (v 1.22.0). Finally, taxonomy assignment was done using the Naive Bayesian Classifier and the DADA2 formatted Silva v.138.1. All statistical analysis was performed in R (v 4.1.3). Potential contaminants were identified and removed with Decontam using a prevalence-based approach (threshold p = 0.5). Microbiota data are inherently compositional, and ASV counts were transformed using the centred log(2)-ratio (CLR) to account for this. A principal component analysis (PCA) was performed on a euclidean distance matrix generated from CLR transformed counts. Significant differences in beta diversity were assessed using permutation multivariate analysis of variance (PERMANOVA) in vegan. Pairwise PERMANOVA comparisons were conducted using the pairwise Adonis package. Alpha diversity was calculated at ASV level and was based on the Shannon index, a robust estimation of both richness and evenness, using the microbiome package. Changes in Shannon diversity over time were assessed using the splinectomeR package. Differential abundance testing was performed using the Negative Binomial and Zero-Inflated Mixed Models (NBZIMM) package.

For manuscripts utilizing custom algorithms or software that are central to the research but not yet described in published literature, software must be made available to editors and reviewers. We strongly encourage code deposition in a community repository (e.g. GitHub). See the Nature Portfolio guidelines for submitting code & software for further information.

Data

Policy information about availability of data

All manuscripts must include a data availability statement. This statement should provide the following information, where applicable:

- Accession codes, unique identifiers, or web links for publicly available datasets
- A description of any restrictions on data availability
- For clinical datasets or third party data, please ensure that the statement adheres to our policy

Data and code availability

The raw data and data frames have been deposited in Mendeley Data, V1, doi: 10.17632/5fgvkb4hyz.1.

Research involving human participants, their data, or biological material

Policy information about studies with human participants or human data. See also policy information about sex, gender (identity/presentation), and sexual orientation and race, ethnicity and racism.

Reporting on sex and gender	NA
Reporting on race, ethnicity, or other socially relevant groupings	NA
Population characteristics	NA
Recruitment	NA
Ethics oversight	NA

Note that full information on the approval of the study protocol must also be provided in the manuscript.

Field-specific reporting

Please select the one below that is the best fit for your research. If you are not sure, read the appropriate sections before making your selection.

☒ Life sciences ☐ Behavioural & social sciences ☐ Ecological, evolutionary & environmental sciences

For a reference copy of the document with all sections, see nature.com/documents/nr-reporting-summary-flat.pdf

Life sciences study design

All studies must disclose on these points even when the disclosure is negative.

Sample size	Pig study n = 30
Data exclusions	NA
Replication	*The challenge model employed is one which have been developed and refined at Aarhus University and published extensively, meaning it is optimized to ensure an as uniforn as possile outcome across trials with the piglets bred in-house, and genetically tested, and a well-known challenge strain.*
Randomization	Pigs (n = 30, initial body weight mean 7.9 kg STD 1.18 kg) from three sows (Yorkshire x Landrace x Duroc) fed a standard Danish sowstarter diet were used in the study, randomly distributed ensuring that litter from each sow is represented across all groups. The sows were tested homozygous carriers of the dominant gene encoding intestinal ETEC F4 fimbriae receptors using competitive allele specific PCR (KASP) analysis of the Mucin 4 gene (VHL Genetics, Netherlands), as were the boars used for insemination of the sows. Thus, piglets were genetically susceptible to ETEC F4+LT+. The piglets were vaccinated against Mycoplasma hyopneumoniae, and the sows against parvovirus, E. coli, and swine erysipelas. Female and castrated males were included in the study. On day 23-26 after birth, piglets of both sexes were weaned, allocated to three experimental groups, balanced according to initial body weight, and housed in pens with two littermates (5 pens per group). Pairs of littermates were housed in the same pen (215 cm x 110 cm), with 75 cm x 110 cm slatted floor, and 140 cm x 110 cm concrete floor with floor heating and partial coverage. Pigs from the BL1.2 +BL2.2 and BL2.2 groups were house in the same room, each on either side of the aisle, and the Control was housed in a similar room. No physical contact was allowed between piglets from different pens. To avoid affecting the gastrointestinal system and experimental parameters, no bedding was allowed, but each pen was provided with a rope, which could help to satisfy the natural behavior of the piglets. The room temperature was 25°C the first week after weaning and then gradually reduced to 21°C the third week.
Blinding	*Groups were not blinded, as the practical circumstances in the experimental barn and the number of trained staff running the trial did not allow this. As we are working with a challenge model, cross-contamination between pens is a concern which needed to be handled.*

April 2023

Reporting for specific materials, systems and methods

We require information from authors about some types of materials, experimental systems and methods used in many studies. Here, indicate whether each material, system or method listed is relevant to your study. If you are not sure if a list item applies to your research, read the appropriate section before selecting a response.

Materials & experimental systems

n/a	Involved in the study
☒	☐ Antibodies
☒	☐ Eukaryotic cell lines
☒	☐ Palaeontology and archaeology
☐	☒ Animals and other organisms
☒	☐ Clinical data
☒	☐ Dual use research of concern
☒	☐ Plants

Methods

n/a	Involved in the study
☒	☐ ChIP-seq
☒	☐ Flow cytometry
☒	☐ MRI-based neuroimaging

Animals and other research organisms

Policy information about studies involving animals; ARRIVE guidelines recommended for reporting animal research, and Sex and Gender in Research

Laboratory animals	Pigs (n = 30, initial body weight mean 7.9 kg STD 1.18 kg) from three sows (Yorkshire x Landrace x Duroc)
Wild animals	NA
Reporting on sex	*Female and castrated males were included in the study to ensure a uniform outcome. On day 23-26 after birth, piglets of both sexes were weaned, allocated to three experimental groups, balanced according to initial body weight, and housed in pens with two littermates (5 pens per group). Sex-based analysis was not performed, as the behavior and development of newly weaned piglets is the same across both sexes.*
Field-collected samples	Throughout the trial faecal samples were collected either from the pen-floor or directly from the rectum of piglets. The samples were moved to eppendorf tubes and immediately put on ice and consequently frozen to -80 degrees Celsius to conserve sample integreti prior to further processing.
Ethics oversight	The in vivo piglet challenge study was carried out at Aarhus University (AU Viborg, Denmark) according to a licence obtained by the Danish Animal Experiments Inspectorate, Ministry of Food, Agriculture and Fisheries, Danish Veterinary and Food Administration (approval no. 2017-15-0201-01270). Animal care and housing were in accordance with Danish laws and regulations governing the humane care and use of animals in research.

Note that full information on the approval of the study protocol must also be provided in the manuscript.

Plants

Seed stocks	NA
Novel plant genotypes	NA
Authentication	NA
